# A focus on the normal-appearing white and gray matter within the multiple sclerosis brain: a link to smoldering progression

**DOI:** 10.1007/s00401-025-02923-1

**Published:** 2025-08-10

**Authors:** Gema Muñoz González, Bert A. ´t Hart, Marianna Bugiani, Jason R. Plemel, Geert J. Schenk, Gijs Kooij, Antonio Luchicchi

**Affiliations:** 1 Department of Anatomy and Neurosciences, Amsterdam University Medical Center, VU Medical Center, VU University, De Boelelaan 1108, 1081 HZ Amsterdam, The Netherlands; 2 Department of Paediatrics and Child Neurology, Emma Children’s Hospital, Amsterdam University Medical Center, Amsterdam, The Netherlands; 3 Amsterdam Leukodystrophy Centre, Emma Children’s Hospital, Amsterdam University Medical Center, Amsterdam, The Netherlands; 4 Department of Pathology, Amsterdam Neuroscience, Amsterdam University Medical Center, Amsterdam, The Netherlands; 5 Department of Medicine, Division of Neurology, Department of Medical Microbiology and Immunology, Neuroscience and Mental Health Institute, Li Ka Shing Institute of Virology, University of Alberta, Edmonton, AB Canada; 6 Department of Molecular Cell Biology and Immunology, Amsterdam University Medical Center, Amsterdam, The Netherlands; 7 Amsterdam Neuroscience, Amsterdam University Medical Center, Amsterdam, The Netherlands; 8 MS Center Amsterdam, Amsterdam UMC location Vrije Universiteit Amsterdam, Amsterdam, The Netherlands; 9 Amsterdam Institute for Immunology and Infectious Diseases, Amsterdam University Medical Center, Amsterdam, The Netherlands

**Keywords:** Smoldering disease, Oligodendrocyte, Myelin, Degeneration

## Abstract

Multiple sclerosis is a chronic neuro-inflammatory and neurodegenerative disease, traditionally characterized by the presence of focal demyelinating lesions in the CNS. However, accumulating evidence suggests that multiple sclerosis pathophysiology extends beyond such classical lesions, affecting also ‘normal’ appearing tissue in both white and gray matter, referred to as ‘normal-appearing white matter’ and ‘normal-appearing gray matter’, respectively. Here, we provide a comprehensive overview of the widespread biochemical, cellular, and microstructural alterations occurring in these ‘normal-appearing’ CNS regions. Additionally, we discuss the evidence derived from human post-mortem studies that support that normal-appearing white and gray matter could be the drivers of smoldering-associated pathological worsening once repair mechanisms are exhausted. Comprehensive understanding of multiple sclerosis pathology beyond classical lesions not only provides a more complete picture of disease progression, but also provides further insights into potential novel therapeutic avenues in order to slow or halt disability accumulation.

## Introduction

**Fig. 1:**
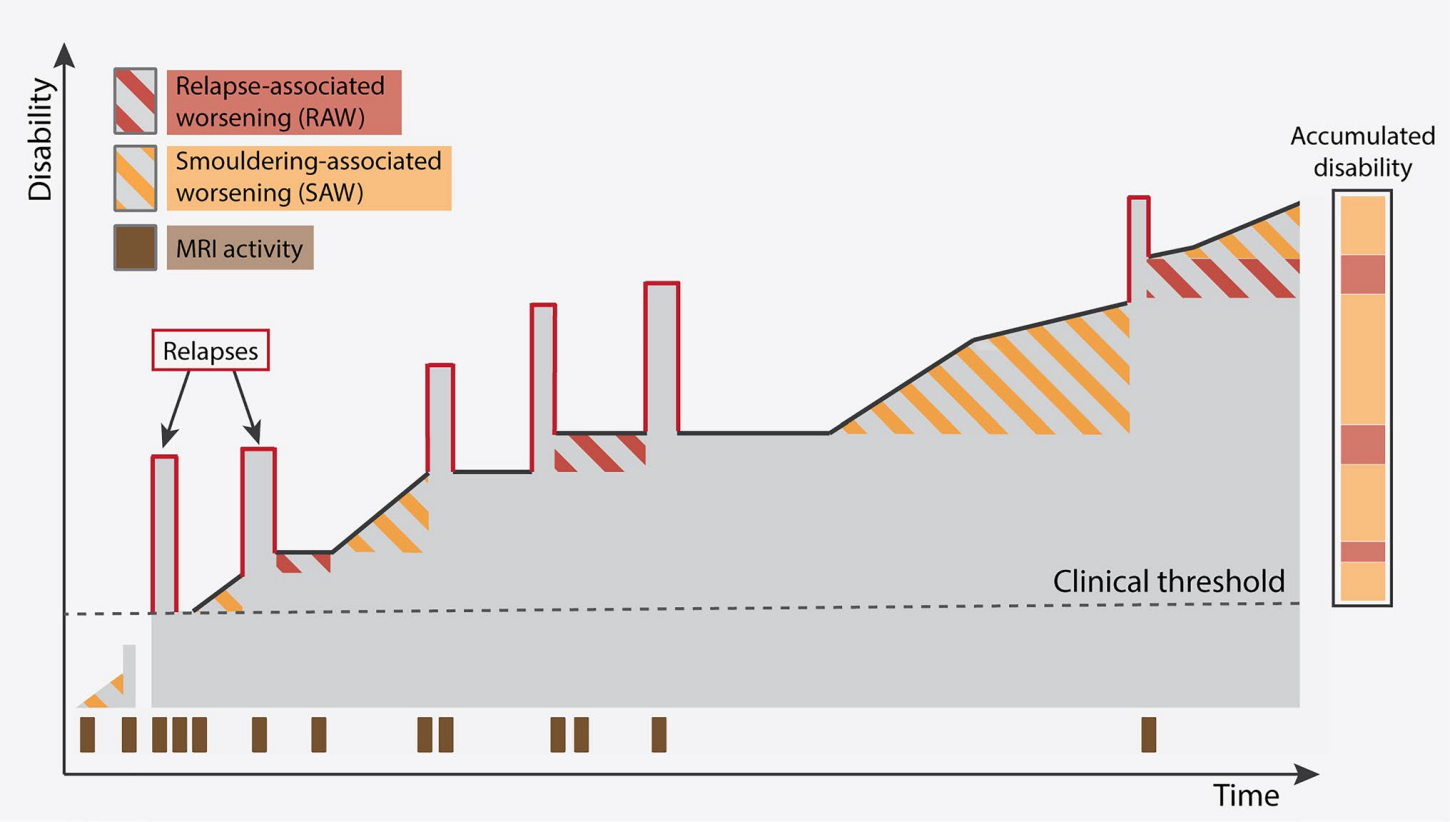
Natural clinical course of Multiple Sclerosis (MS). Relapse-associated worsening (RAW) is more frequent during the early phases of MS but decreases as the disease progresses. In contrast, smoldering-associated worsening (SAW) gradually accumulates and becomes more noticeable over time, particularly as relapses diminish. SAW is the primary driver of disability accumulation, even in the early stages of the disease

Multiple sclerosis (MS) is a chronic neuro-inflammatory and neurodegenerative disease of unknown etiology [82]. The classical pathological hallmarks are demyelinating lesions, defined as a focal area of myelin loss with a variable degree of inflammation, astrogliosis, and neuronal degeneration [105]. Lesions can be found in white matter (WM) as well as gray matter (GM) of the brain and the spinal cord although they differ with respect to histological phenotype and time of appearance [105]. Within lesions, histological examinations show a variety of immune cells, suggesting that the damage in MS is at least partly driven by peripherally activated immune cells infiltrating the central nervous system (CNS) and attacking myelinated axons. With these observations in mind, a plethora of disease-modifying therapies aimed at dampening the immune system have become available [70]. Even though these interventions are highly efficacious at suppressing both the formation of new lesions and clinical relapses, they minimally impact the relentless clinical progression observed [218]. Progression independent of immune-driven relapses is termed either smoldering-associated worsening (SAW) or smoldering disease when patient-reported outcomes are used, or progression-independent of relapse activity (PIRA) when the clinical score EDSS is specifically used [174]. SAW is a major driver of disability accrual throughout the disease course [111, 161, 201], even as early as after the first demyelinating event [86, 87]. Thus, two parallel pathogenic events coexist in MS: (1) a progressively accumulating degenerative process that persists throughout all phases of disease, and (2) superimposed bouts of inflammatory activity that can be detected with MRI and are confined to the relapsing phase (Fig. 1). These and other observations conflict with the historical lesion-centered paradigm of MS pathology, which posits that halting lesion development is sufficient for stopping clinical worsening. Instead, effective therapies likely need to also target the smoldering pathology outside the acute lesion in order to limit progression [174].

**Fig. 2:**
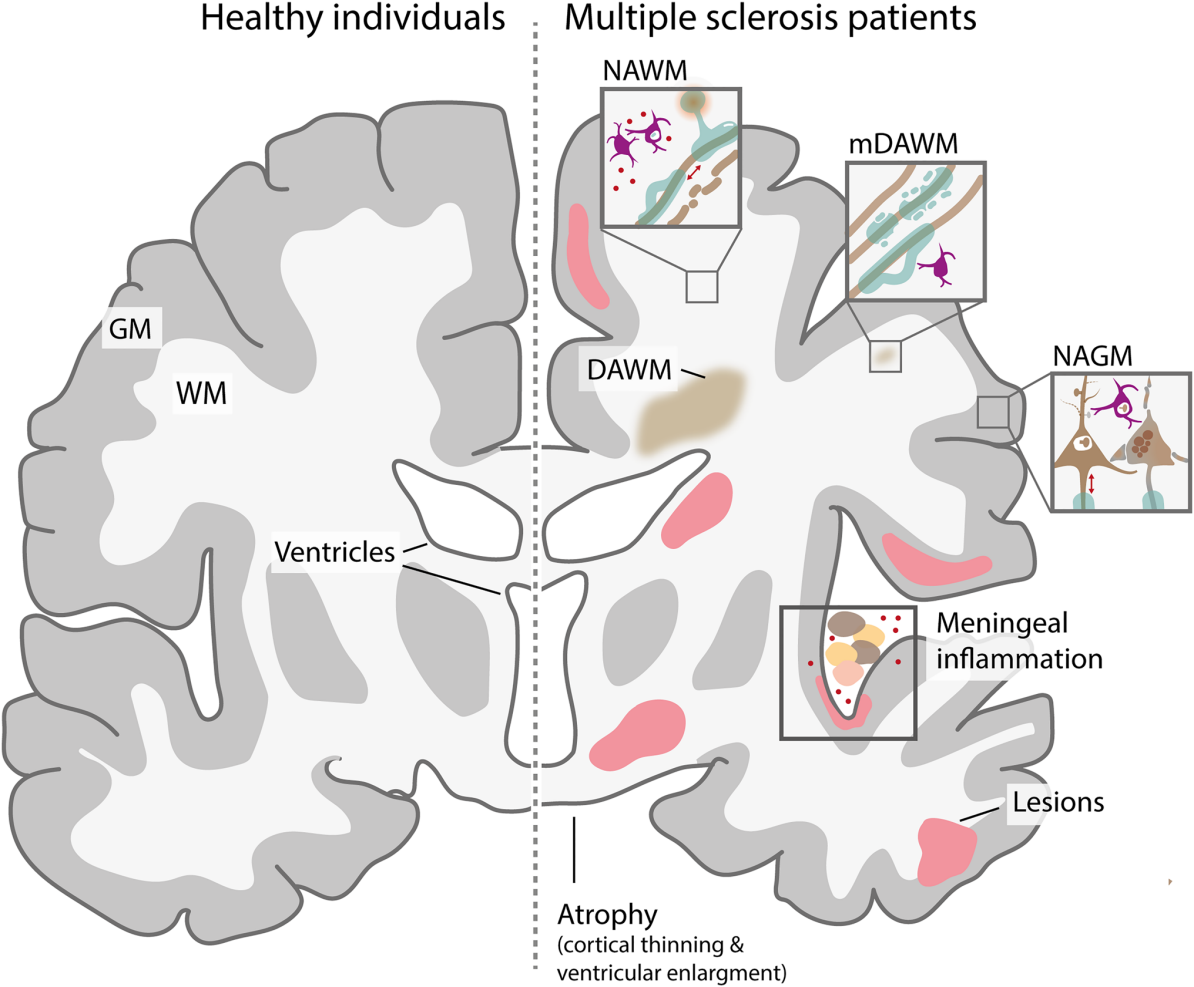
Pathological correlates in Multiple Sclerosis (MS) compared to control CNS tissue. Key pathological changes in MS, including alterations in normal-appearing white matter (NAWM) and normal-appearing grey matter (NAGM), diffusely abnormal white matter (DAWM) and micro-diffusely abnormal white matter (mDAWM), meningeal inflammation-associated damage, atrophy, and MS lesions

The reconsideration of the lesion-centered concept of MS draws attention to the less-studied pathogenic processes in the normal-appearing white and gray matter outside demyelinating lesions (Box 1 and Fig. 2). Compared with WM from non-neurological controls, normal-appearing white matter (NAWM) from people with MS displays, among others, reduced density of specific oligodendroglia populations, aberrant myelin structure and composition, and exacerbated microglial reactivity. In the normal-appearing gray matter (NAGM) of people with MS, myelin disturbances accompany the loss of particular neuronal populations, synaptic demise, and microglial alterations compared with the GM of non-neurological controls. A deeper understanding of the “normal-appearing” areas in MS can not only provide a more comprehensive insight into the disease etiology, but also guide therapy development. With this goal, this review gives a concise though exhaustive literature summary of the main players in this pathogenic process: oligodendrocytes, microglia, astrocytes, neurons, and lymphocytes. Based on the evidence presented here, we consider of paramount importance to review the long-standing dogma that posits lesioned areas as the sole or most-important pathological correlate of MS and consider normal-appearing tissue also as a pathological entity.

Box 1 Pathological entities in multiple sclerosis beyond lesions Despite lesions have been traditionally considered the hallmark of multiple sclerosis (MS), it is increasingly recognized that pathological processes that possibly contribute to disease progression extend beyond focal lesions (Fig. 2). Importantly, the different pathological entities should not be considered segregated, as likely they are interacting processes.

Normal-appearing white matter (NAWM): Regions that appear unaltered on conventional MRI, and are located at least 1 cm from visible lesions. Histopathological analysis reveals an absence of confluent demyelination, but numerous signs of tissue damage, including subtle myelin loss, axonal transection and degeneration, as well as widespread microglial activation accompanied by perivascular T cell infiltration. Quantitative MRI shows diffuse myelin alterations and microglial activation in these regions even in early disease stages [44, 62].

Normal-appearing gray matter (NAGM): Areas outside lesions that appear normal on conventional MRI and in which no confluent demyelination is present, but exhibit diffuse alterations, such as widespread synapse loss, axonal demise, and subtle signs of neuronal damage.

Micro diffusely abnormal white matter (mDAWM): Microscopic regions within NAWM that appear normal on MRI but show myelin pallor and signs of myelin instability (such as increased citrullination of myelin basic protein and elevated frequency of myelin blisters [114]) on histological staining [114]. Despite being mostly located around central veins, mDAWM areas do not show T or B cell infiltration [114].

Diffusely abnormal white matter (DAWM): Large regions of white matter that exhibit a proton density and T2-weighted MRI signal intensity intermediate between NAWM and lesions. DAWM regions are often in located in periventricular areas or the centrum semiovale, and are present in approximately 25% of people with MS [212, 214, 216]. Histopathological analyses reveal partial myelin phospholipid loss (with relatively preserved myelin proteins), axonal degeneration, astrocytosis, and blood–brain barrier breakdown (reviewed in Ref. [21]). Although sometimes DAWM occurs in the vicinity of a lesion, it is not always the case [61]. While it has been suggested that DAWM represents secondary degeneration to distal focal lesions, recapitulating a possible manifestation of Wallerian degeneration [178], its early appearance in MS is suggestive of an underlying primary oligodendrocyte pathology [75].

Leptomeningeal inflammation: accumulation of immune cells—such as B cells, T cells, and myeloid cells—within the subarachnoid space, either diffusely scattered or forming aggregates resembling tertiary lymphoid follicles. Leptomeningeal inflammation can be detected through post-mortem histology or advanced imaging techniques [69] and is particularly prevalent in progressive MS, affecting approximately 40% of patients [118]. Meningeal inflammation strongly correlates with local cortical pathology [77, 119, 211], which is likely driven by the release of pro-inflammatory cytokines. Importantly, this pathological feature is not specific to MS; it also occurs in other neuro-inflammatory and infectious diseases and, albeit less commonly, in healthy individuals [1].

Whole brain atrophy: brain atrophy begins early during MS and continues to progress throughout the disease. On T1-weighted MRI, it is characterized by the widening of cortical sulci and enlargement of the ventricles. Ventricular enlargement, which is indicative of MS-specific white matter atrophy and not typically seen in age-related atrophy [151], begins in the early disease stage [151, 159]. In contrast, cortical atrophy generally becomes apparent later [151, 159].

## Myelin pathology and oligodendroglia dysfunction: a smoking gun

The myelin sheath is wrapped around axons, facilitating fast and energy-efficient axonal signal transmission [165] and avoiding signal interference between contiguous axons running in parallel [177]. Beyond its classic role as a passive insulator, myelin also metabolically supports the enwrapped axon with monocarboxylates (lactate/pyruvate [170] and glucose [131]) via the so-called axo-myelinic synapse [188]. Additionally, myelin participates in the siphoning of K^+^ accumulating in the periaxonal space upon high electrical activity [117]. Thus, WM myelin ensures that information is efficiently propagated, acting as an insulator, but also facilitating the restoration of axonal membrane potentials through metabolic support and K^+^ dispersal. In the GM, myelin coverage is scarcer and less regular than in the WM, with many neurites containing only a single myelinated segment [186, 197]. Although this distinctive myelination pattern is unlikely to notably accelerate impulse conduction [12, 197], GM myelin possibly contributes to metabolically support neurites [98, 133], fine-tune network function by subtly modulating conduction speed [179], and prevent aberrant dendrite sprouting [52]. However, much less is known about the functions of GM myelin compared to its WM counterpart (reviewed in Ref. [195]).

### Aberrant myelin structure within NAWM

Myelin loops are anchored to the axon they ensheath through transmembrane adhesion proteins located at the periphery of each myelin segment, a region called the paranode. These axo-myelin paranodal contacts establish a physical boundary that orchestrates the regional specialization along myelinated axons. The non-myelinated segments, known as the Nodes of Ranvier, are rich in voltage-gated Na^+^ channels (Na_v_), while the myelinated regions immediately adjacent to the paranodes, termed juxtaparanodes, are enriched in voltage-gated K^+^ channels (K_v_) [3]. Nodal and paranodal structures are critical for saltatory conduction, and even minor disruptions to their organization can alter action potential propagation [4]. Additionally, the axo-myelin paranodal contacts regulate both myelin thickness and nodal length, as when these contacts are loosened, the outermost myelin layer is filled with cytoplasm and eventually retracts, reducing myelin thickness and increasing nodal length [48].

As in MS lesions [223], nodal, paranodal, and juxta-paranodal regions are disorganized in MS NAWM (reviewed in Ref. [3]). NAWM regions of both optic nerve and the cerebrum of people with MS show increased paranodal length [59, 78, 113, 205] and protrusion of juxta-paranodal Kv1.2 channels toward the paranode [59, 89, 205]. Mathematical simulations indicate that paranodal lengthening and juxtaparanodal K^+^ channel dislocation compromises action potential propagation, especially in small diameter fibers [59]. Furthermore, the K⁺ channel Kir4.1, localized in the inner and outer myelin leaflets in paranodal regions, is downregulated in MS NAWM compared to control WM [85], possibly contributing to alterations in action potential propagation.

The disruption of the archetypical regional organization along myelinated axons in MS may result from the detachment of myelinic and axonal adhesion proteins at the paranodes. Several paranodal axo-glial tethering proteins, including contactin-associated protein 1 (Caspr1) and contactin 1, exhibit an extended expression pattern along MS NAWM axons [59, 113, 205], possibly due to their mutual detachment and subsequent diffusion along the axonal and myelin membranes. Alterations in the paranodal regions of MS optic nerves are accompanied by signs of compromised myelin integrity (namely, reduced myelin compaction and increased g-ratio, which refers to the relation between the diameter of the axon to the outer diameter of the entire myelinated fiber) [205]. This supports a scenario in which axo-glial contacts are partly lost and the outermost layers are filled with cytoplasm, but have not yet retracted.

The cause of a potential axo-glial detachment in MS remains unclear. Under physiological conditions, axo-glial interactions are maintained by perinodal astrocytes, which secrete a thrombin inhibitor preventing their disruption [48]. Although it is not yet known whether this regulatory mechanism is compromised in MS, thrombin levels are elevated in plasma of people with relapsing–remitting MS [65, 154]. Compelling evidence suggests that immune cells may also contribute to the axo-glial detachment in MS [59, 205]. In normal-appearing optic nerves, paranodal elongation, juxtaparanodal Kv1.2 displacement, and myelin ultrastructural abnormalities correlate with density of T lymphocytes and reactive microglia in the surrounding areas [205]. In vitro studies using murine cerebellar organotypic brain slices demonstrate that treatment with pro-inflammatory cytokines (tumor necrosis factor, TNF, and interferon gamma) can induce paranode elongation [59], suggesting that reactive microglia, astrocytes, and T cells possibly secrete toxic molecules that contribute to the observed paranodal changes. However, unmasking of Kv1.2 channels at the juxtaparanodes or the downregulation of Kir4.1 at the vicinity of Nodes of Ranvier is expected to elevate extracellular K^+^ concentration, which would in turn trigger the activation of adjacent microglia [167]. Thus, it remains difficult to determine whether paranodal disruptions or immune cell reactivity occur as the cause or consequence of one another although it is likely that both mechanisms reinforce each other in a vicious cycle.

In contrast to the findings in optic nerves, no significant signs of myelin decompaction are found between NAWM in the MS brain and normal WM in healthy controls [147]. However, brain NAWM of patients with progressive MS shows a global reduction in myelinated fibers with a shift in the distribution of the axon radii of the myelinated fibers toward larger axons [147]. Oost et al*.* provide a plausible explanation suggesting that while the smallest NAWM axons are lost, the spared axons swell, and the thickness of myelin is increased to maintain conduction velocity (and thus, the g-ratio remains unchanged) [147]. Given that incorporation of new myelin occurs mainly at the paranodal region [129], the disorganization of nodal and paranodal areas in MS NAWM could stem from the proposed adaptive myelination upon axonal swelling. Further supporting the occurrence of axonal swelling in MS NAWM, a diffuse increase in mean axonal caliber in NAWM areas from people with MS compared to WM from healthy controls was detected using an MRI proxy [30].

**Fig. 3:**
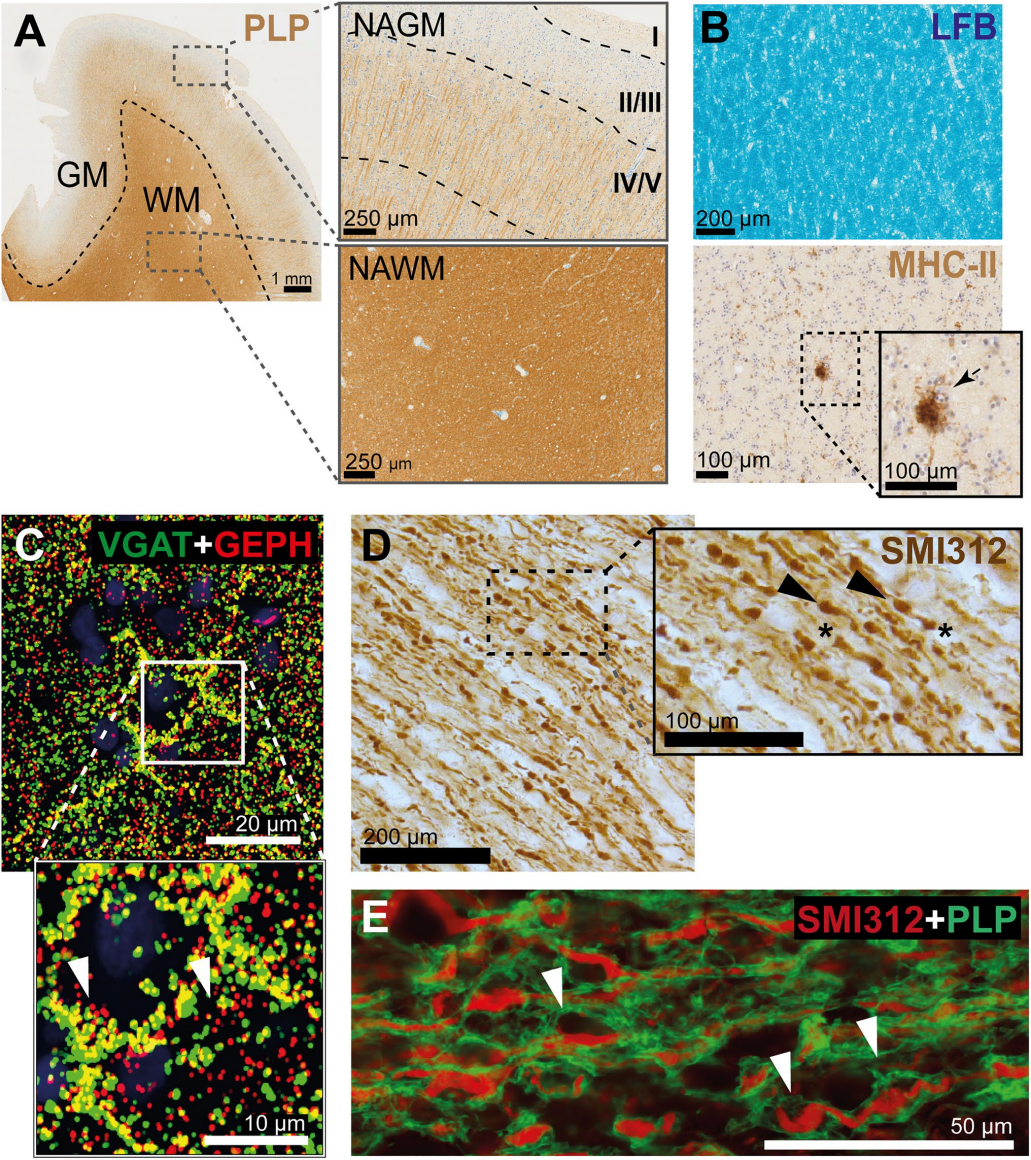
Pathological alterations in normal-appearing gray matter (NAGM) and white matter (NAWM) in Multiple Sclerosis (MS). **A** Representative image of an MS brain tissue section stained with an antibody against PLP, highlighting regions of both NAGM and NAWM. **B** Luxol Fast Blue (LFB) staining (top) reveals compact and homogeneous lipid distribution within NAWM. In the same region, staining for MHC-II (bottom) shows microglial clustering. **C** Staining for vGAT (a marker of inhibitory presynaptic terminals) and Gephyrin (a postsynaptic inhibitory marker). Co-localization (yellow) alternates with areas where only postsynaptic Gephyrin is detected, indicating loss of inhibitory presynaptic terminals.** D** SMI312 staining in NAWM highlights axonal transections (*) and swellings (arrow bars), reflecting ongoing axonal degeneration.** E** Dual staining for PLP and SMI312 in NAWM reveals local detachments of the myelin sheath from the underlying axons (“myelin blisters”)

That myelin in MS also undergoes decompaction or swelling is supported by the observation of local myelin detachments (termed “myelin blisters” or “myelin swellings”) in NAWM (Fig. 3E) [113] and of local myelin outfoldings at the adaxonal surface (“myelinosomes”) in both NAWM and lesions [166]. Both myelin blisters and myelinosomes are pathological entities thus far specific of MS: myelin blisters are more frequent in MS than in non-demented controls and patients with Alzheimer’s disease or encephalitis [113], while myelinosomes are absent in controls and patients with progressive multifocal leukoencephalopathy [166]. Notably, local myelin swellings in MS differ from those observed in non-neurological controls in both regional distribution and chemical composition [113]. In control WM, myelin swellings predominantly locate in perinodal regions, whereas in MS NAWM, they are distributed evenly throughout the myelin sheath [113]. Local blister-like myelin detachments reminiscent of those in MS tissue are an early pathological feature observed in cuprizone-fed mice [83], an animal model in which oligodendrocyte death and demyelination are induced by the copper-chelator cuprizone. Cuprizone-induced myelin detachments are primarily observed around the internodes [83] similar as those in patients with MS [113]. Altogether, these findings suggest that oligodendroglia dysfunction may underlie internodal myelin blistering in MS, which could precede myelin loss. Consistent with this hypothesis, we recently identified distinct foci of subtle myelin pallor and increased blister frequency within MS NAWM, which we termed micro-diffusely abnormal white matter (mDAWM) [114]. It is also noteworthy that in cuprizone-fed mice, myelin swellings are mainly located to small-caliber axons [83], which are the first to be lost in MS NAWM [147]. These axons are particularly vulnerable to energy deprivation due to their high surface-to-volume ratio and, thus, heavily rely on metabolic support from myelin. The increased distance between the myelin and the axon as a result of myelin blistering may impair the shuttling of energy-rich substrates, potentially contributing to axonal degeneration, especially of small axons [113]. Furthermore, since the periaxonal space contributes to the saltatory conduction along myelinated axons [40], the enlargement of this space due to myelin blistering may also disrupt axonal signal conduction.

### Myelin composition is altered in NAWM and NAGM

The structural properties of myelin stem from its biochemical composition. Compared to other biological membranes, myelin exhibits a higher lipid-to-protein ratio, with lipids comprising 70–85% of its composition, compared to the typical 1:1 lipid-to-protein ratio found in most cellular membranes. Additionally, myelin contains a distinct abundance of specific lipid families, with particularly high amounts of cholesterol and glycolipids. Among proteins, proteolipid protein (PLP), myelin basic protein (MBP), 2',3'-cyclic nucleotide 3'-phosphodiesterase (CNPase), myelin oligodendrocyte glycoprotein (MOG), and myelin-associated glycoprotein (MAG) account for most of the total protein mass. Such a specific protein and lipid composition is crucial for ensuring tight interactions between the apposed lamellae, which are bound together through non-covalent connections between proteins and lipids. Even minor alterations in myelin constituents can increase local myelin curvature, causing structural aberrations and myelin instability [181], which potentially compromise its functions.

Regions of swollen myelin in MS NAWM present a different biochemical fingerprint than swollen myelin segments in WM from non-neurological controls, based on Coherent Anti-Stokes Raman Scattering (CARS) imaging [113]. Specifically, MS swellings show a decrease of the vibrational spectrum corresponding to CH_2_ bonds, indicative of a putative reduction in long-chain fatty acids [113]. Another CARS study revealed widespread spectral differences in normal-appearing corpus callosum myelin from people with MS, indicating that changes in the myelin lipid biochemistry and molecular order may extend beyond locally swollen areas [160]. Using the solvatochromic dye Nile Red, investigation of myelin polarity revealed heterogeneity within NAWM [193]. Some myelin segments in NAWM exhibit higher polarity than the uniformly low polarity observed in controls, resulting in an overall higher polarity index compared to control WM myelin [193]. The observed increased polarity could either be due to a relatively higher proportion of polar lipids or to increased myelin water content from widened lamellae, as also suggested by diffusivity imaging studies [135]. Increased polarity due to any of these reasons would increase myelin capacitance and is predicted to functionally impair axonal conduction velocity [193]. Interestingly, cuprizone intoxication in mice leads to increased myelin polarity in the corpus callosum as early as two days after the start of the diet, further supporting the idea that elevated myelin polarity is an early sign of myelin damage preceding demyelination [193]. In sharp contrast with NAWM, NAGM of people with MS presented a reduced myelin polarity compared to non-demented controls [193]. Albeit speculative, such changes may reflect subtle adaptive myelin modifications to modulate circuit activity, potentially serving as a compensatory mechanism to counteract some of the neuronal alterations observed in NAGM, such as synaptic loss.

Further supporting an altered myelin composition in MS, mass spectrometry analysis revealed increased sphingolipid and decreased phospholipid and cholesterol content in MS NAWM compared to the WM of non-neurological controls [221]. Computational modeling predicts that these changes may increase the repelling force between the opposing myelin bilayers, reducing myelin compaction [221]. However, in this study, lipid species were measured on bulk tissue samples, and thus the results cannot be solely attributed to myelin as they were likely influenced by other sources of lipids. Along the sphingolipid and phospholipid alterations, NAWM (but not NAGM) presented large accumulations of derivatives of the lipid peroxidation end-product 4-hydroxynonenal (4-HNE) [221]. Lipid peroxidation is a cyclic reaction in which free oxygen radicals, such as hydroxyl radical (HO•), attack unsaturated bonds (-C = C-) in polyunsaturated fatty acids that are either in their free form or esterified as phospholipids. Peroxidation of membrane lipids results in the truncation of phospholipids at the sn-2 site, forming oxidized phospholipids and lipid hydroperoxides, which are unstable and decompose into a series of stable aldehydic products, such as 4-HNE. Truncation of polyunsaturated fatty acids within cellular membranes increases their instability and permeability [9, 215], and accumulation of 4-HNE can alter membrane fluidity and affect phospholipid asymmetry [29]. Thus, increased lipid peroxidation likely results in increased myelin polarity, not only due to the addition of -OOH groups, but also due to increased membrane fluidity stemming from phospholipid truncation and 4-HNE accumulation. Incubation of myelin fractions with oxidants in vitro resulted in decompaction of the intraperiodic line of myelin [18], consistent with lipid peroxidation weakening the adhesion between myelin lamella. This effect however was probably not solely due to lipid peroxidation, but also partly due to the oxidation and subsequent loss of tertiary structure of PLP.

Myelin proteins are also altered in MS NAWM. MBP, a protein essential for anchoring the cytoplasmic leaflets of myelin lamellae, undergoes extensive citrullination in MS NAWM [113]. Protein citrullination (or deimination) is a posttranslational modification catalyzed by the Ca^2+^-dependent enzyme protein arginine deiminase (PAD), wherein positively charged arginine residues are converted into the neutral amino acid citrulline. Citrullination weakens the interaction between MBP and the negatively charged phospholipids of myelin, and promotes MBP unfolding, cleavage, and exposure of the immunodominant epitopes (as reviewed elsewhere [230]). Several studies have reported increased expression levels and activity of the predominant PAD isoform in the CNS (PAD2) in NAWM myelin [124, 137, 227], a finding that may be explained by the hypomethylation of the PAD2 promoter [124]. Of note, increased citrullination of MBP is accompanied by reduced phosphorylation and increased methylation [96] although the functional relevance (if any) of such posttranslational modifications remains to be established.

In conclusion, myelin from NAWM presents a plethora of alterations ranging from composition to structure, potentially leading to decreased myelin stability and/or impaired functionality [59, 193, 221]. As in vitro data obtained using myelin-like multilamellar vesicles demonstrate that composition alterations can cause aberrant membrane structure [181], it is tempting to speculate that lipid and protein disturbances underlie some of the structural myelin aberrations observed in MS, such as myelin blisters, myelinosomes, or paranodal alterations, and ultimately make myelin more prone to degradation. Interestingly, MS-derived myelin is phagocytosed more efficiently by macrophages and microglia than myelin derived from controls [73]. Furthermore, in vivo studies confirm that subtle biochemical changes of myelin are sufficient to elicit a demyelinating inflammatory immune response [26], further positioning biochemical and structural myelin alterations as drivers of myelin loss.

### Oligodendroglia skewing accompanies myelin alterations

Since oligodendrocytes were discovered by Rio-Hortega in the 1920s, their remarkable morphological heterogeneity was recognized [76]. However, oligodendroglia heterogeneity and its potential functional significance have remained largely unexplored until development of single-cell RNA sequencing technologies. We now know that distinct oligodendrocyte subtypes with unique transcriptional signatures and preferential localizations within the CNS exist both in mice [53, 120, 122] and humans [81]. For example, the human oligodendrocyte subtype termed “Oligo6” expresses high levels of Opalin and localizes to the junction between GM and WM. In MS NAWM, the balance between the eight subpopulations of mature oligodendrocytes (reported) in the healthy mature human brain is altered [81]. Compared to control WM, the most mature oligodendrocyte subpopulation (“Oligo1”) and the intermediate population (“Oligo6”) are profoundly reduced in MS NAWM. By contrast, other subpopulations prevail in NAWM compared to WM from controls, such as “Oligo2”, which displays a stress response signature. Further supporting that oligodendrocytes in MS may be under stress conditions, oligodendrocytes from NAWM and MS lesions accumulate stress granules (condensates of ribonucleoproteins and messenger RNAs stalled in translation) compared with oligodendrocytes from control WM [155].

The regeneration of myelin, or remyelination, was traditionally considered to require oligodendrocyte progenitor cells (OPCs) differentiation. In MS, remyelination is thought to occur in shadow plaques, which have an intermediate lipid staining. However, carbon-dating analyses on human individuals revealed that newly generated oligodendrocytes are absent within remyelinated (shadow) plaques of most people with MS [233]. Studies in animal models have shown that pre-existing oligodendrocytes can produce new myelin segments [10, 47, 141] although this type of remyelination is often defective [141]. This suggests that impaired OPC-mediated remyelination within shadow plaques may be partially compensated by remyelination driven by mature oligodendrocytes. However, this compensatory mechanism may be insufficient when large areas of demyelination occur, as seen in classic lesions.

Myelin repair and regeneration by both OPCs and mature oligodendrocytes may also occur in NAWM. Although NAWM does not exhibit confluent demyelination, subtle myelin loss and widespread myelin structural abnormalities occur [104, 147, 205]. Genes encoding MAG, PLP, or CNP are upregulated in mature oligodendrocytes within NAWM compared to those in WM from controls [81], suggesting that new myelin sheaths are generated or repaired in the NAWM by pre-existing oligodendrocytes. New sheaths may arise through de novo myelination as a part of myelin plasticity, common in animal models [126, 152, 185, 228]. Alternatively, lost myelin may be regenerated through remyelination. While single-cell transcriptomic data indicate that OPCs are less abundant in MS NAWM than in control WM [81], carbon-dating studies reveal that oligodendrocyte turnover in NAWM from a subset of MS patients is approximately threefold higher than in WM from healthy individuals [233]. Thus, it is likely that, at least in a subset of MS patients, remyelination by newly differentiated oligodendrocytes arising from OPCs occurs in NAWM, with the new myelin becoming indistinguishable from the original. Supporting this, studies in zebrafish have shown that the regeneration of myelin sheaths of normal length and thickness is accompanied by axon caliber enlargement [88], a feature that is also observed in NAWM [147]. Notably, no significant differences in gene expression were observed in the NAWM between efficiently and poorly remyelinating MS donors [33], suggesting that the subtle remyelination occurring in NAWM may take place in both good and poor remyelinating donors, and that the determinants of remyelination success may only become apparent in the context of more extensive damage, such as that seen in lesions. In addition to new myelin sheath production, repair of myelin structural disruptions such as myelin decompaction or blistering may also occur within NAWM, although evidence for this is lacking.

Despite the central role of oligodendroglia in MS pathology, there remains a surprising scarcity of studies investigating these cells in normal-appearing tissue. Understanding the distinct routes to oligodendrocyte and myelin damage and their repair or regeneration has critical implications for the development of remyelination-supporting therapies, which currently focus solely on boosting the classical OPC pathway.

## Microglia: the first to fall after myelin damage

Microglia are the predominant innate immune cell type residing within the CNS. In the adult brain, microglia constantly survey their surroundings through motile ramifications and filopodia [13, 143], establishing contacts with diverse neuronal compartments (including synapses [11], soma [41], axon initial segment [8], and nodes of Ranvier [152]), which reciprocally regulate neuronal activity [11] and microglial motility [109, 140]. Microglia also play a critical role in maintaining proper myelin integrity by preventing abnormalities as outfoldings and inner tongue decompaction although the underlying mechanisms remain partially ununderstood [128]. Upon encountering cues of disturbed homeostasis, microglia proliferate [192] and adopt heterogeneous specialized states primarily aimed to combat damage [72]. In the context of myelin damage, microglia clear myelin debris and contribute to OPC recruitment, creating a favorable environment for remyelination [7, 101]. However, if their functions become dysregulated, they may instead exacerbate damage [128]. Outside the lesion environment in MS, both in NAWM and NAGM, microglia exhibit signs of dysregulation, suggesting their involvement in smoldering pathological processes.

### Loss of regional-specificity and metabolic dysregulation in NAWM and NAGM microglia

Similarly to oligodendrocytes, microglia present distinct transcriptional and morphological features in different brain regions, which likely reflect the specific functions they assume in response to environmental demands. In the adult healthy human brain, GM microglia express molecules involved in complement pathway and cytokine-mediated signaling, possibly contributing to synapse pruning and viral defense, respectively [208]. By contrast, WM microglia express molecules mainly related to chemotaxis (supporting environmental surveillance [208]) and to inhibition of inflammatory responses [208] (to maintain a preactivated, rather than fully activated state [236]). Microglia from NAWM and NAGM in people with MS partly lose their region-specific signature: whereas microglia from non-neurological controls exhibit 454 differentially expressed genes between GM and WM, only 125 genes are differentially expressed between NAGM and NAWM microglia in people with MS [208]. Despite this loss of regional identity, microglia from normal-appearing areas retain a homeostatic transcriptome, suggesting that this regional homogeneity is not driven by a general shift toward a reactive, inflammatory state [208]. Instead, distinct patterns of metabolic dysregulation are observed within these regions. While NAWM microglia upregulate genes related to lipid storage and metabolism similarly to microglia within WM lesions, NAGM microglia show increased expression of genes involved in iron homeostasis [208]. In contrast to these findings, another study reported a reduction of the homeostasis markers P2RY12 and TMEM119 and upregulation of genes related to phagocytosis and antigen presentation in NAWM microglia [236]. The discrepancy in microglial activation states observed across studies may arise from differences in the MS cohorts examined. For instance, van der Poel et al. [208] analyzed patients with long-standing disease, whereas Zrzavy et al. [236] focused on individuals with acute MS, in whom a predominantly proinflammatory microglial profile is expected. Differences in transcriptomic technologies may also contribute: Zrzavy et al. [236] used microarrays, while van der Poel et al. [208] employed next-generation sequencing. Although next-generation sequence technologies offer greater sensitivity and dynamic range, subtle variations in transcriptomic analysis pipelines can greatly bias results, underscoring the importance of establishing standardized workflows. Finally, microglial states may also be influenced by comorbid conditions present at the time of death, such as proteinopathies or sepsis, which can modulate microglial activation independently of MS. Future studies should rigorously control for these confounding factors to accurately characterize microglial phenotypes and elucidate MS-specific pathological processes.

### Microglia clustering within NAWM

Besides these phenotypic alterations, MS NAWM consistently shows widespread microglia clustering regardless of disease subtype [209]. Microglia clusters, or nodules, consist of bushy formations of 4–50 HLA-DR-positive microglia (Fig. 3B). They are found in NAWM without blood brain barrier disruption or astrogliosis, and show no clear vascular association [209]. Traditionally considered to represent the earliest stage of lesion formation, microglia nodules are also referred to as ‘type 1 lesions’ or ‘pre-plaques’ [173], ‘preactive lesions’ [204], ‘primordial lesions’ [60] or ‘newly forming lesions’ [203]. However, given their high abundance throughout the brain parenchyma compared to the substantially lower number of demyelinated lesions, most nodules likely resolve or stagnate rather than progress into full-blown demyelinating lesions. Microglia nodules are present in most, but not all people with MS (64% according to [206]). Patients with nodules have a higher lesion load, more active lesions, and fewer inactive lesions than those without nodules [206]. Thus, the absence of nodules may reflect the cessation of new lesion formation, further supported by the early observation that nodule frequency decreases with prolonged disease duration [104, 173].

Microglia within nodules express elevated levels of both pro-inflammatory (e.g., TNF) and anti-inflammatory (e.g., interleukin-10) mediators [209]. They also express the nicotinamide adenine dinucleotide phosphate (NADPH) oxidase complex, an enzyme responsible for producing the reactive oxygen species (ROS) superoxide anion (O₂•⁻) and hydrogen peroxide (H_2_O_2_) [209]. This suggests that microglial nodules may create a pro-inflammatory, ROS-rich microenvironment that can damage both myelin and oligodendrocytes as shown in vitro [136]. Consistent with these potential deleterious effects, nodules are often associated with αΒ-crystallin (CRYAB)-positive oligodendrocytes and partially demyelinated axons [210]. CRYAB is a chaperone protein with anti-inflammatory and neuroprotective effects in the context of myelin damage [149], but it can also elicit strong T cell proliferation and a robust microglial response [19]. Thus, whether oligodendroglial CRYAB accumulation contributes to microglia clustering or whether it is a consequence thereof remains to be determined. On one hand, it is plausible that CRYAB expression is a compensatory mechanism to intrinsic oligodendrocyte stress that triggers secondary microglia clustering [19]. On the other hand, CRYAB expression by oligodendrocytes may reflect a protective response against the stress conditions generated by the neighboring microglial nodule. Nodules in people with MS can also be associated with axons undergoing Wallerian degeneration [182]. While some propose that degenerating axons may trigger microglial clustering [182], others dispute this hypothesis, by pointing at the round shape of microglial clusters—rather than an elongated form—as well as the absence of degenerating axons within the core of many nodules [203].

Microglia nodules are not exclusive to MS; they are also observed in cases of viral encephalitis [34], traumatic brain injury [182], stroke [132] or COVID-19 infection [106]. However, microglia nodules in MS NAWM exhibit distinct features [206]. RNA sequencing of laser-microdissected nodules revealed that genes related to lesion formation (as *HLA-DRB5* and *ISG15*) and MS susceptibility (*IFNAR2*) were selectively upregulated in MS nodules compared to stroke nodules. Additionally, MS nodules showed increased expression of lipid processing enzymes and higher levels of oxidized lysosomal phospholipids than stroke nodules [206]. Importantly, lipid-related alterations were confined to the nodules and absent in non-nodular microglia within the surrounding NAWM [206]. This indicates that lipid dysregulation is not a widespread consequence of diffuse chronic tissue inflammation in NAWM, but rather a nodule-specific phenomenon. Finally, MS nodules often presented a tubular mitochondrial network, indicative of a hypermetabolic and hyperinflammatory state possibly related to clearance of myelin debris [206].

### Microglia-mediated cortical inflammation

Compared to WM areas, GM microglia are scarcer and potentially less reactive in the context of MS. Supporting this, GM lesions exhibit fewer microglial cells than WM lesions [157], although their reactivity is influenced by age and disease duration [100]. Nonetheless, NAGM contains an increased density of CD68^+^ microglia—CD68 being a lysosomal marker commonly used as an indicator of microglia reactivity—compared to control GM [144, 231]. This heightened reactivity in MS NAGM may be driven by the observed concomitant reduction of the checkpoint molecule CD200 in NAGM, which normally maintains microglia in a homeostatic state [207]. Heightened microglia density and reactivity are particularly prominent in the NAGM cortical layers I–III of MS cases with lymphoid follicle-like aggregates in the overlaying meninges [119]. In progressive MS, early stages of meningeal inflammation are marked by high densities of MHC-II^+^- and CD68^+^-microglia in the underlying cortex, which frequently interact with neuronal somata and engage in phagocytosis of presynaptic terminals [211]. By contrast, chronic stages of meningeal inflammation are associated with hyper-ramified microglia with reduced expression of the homeostatic markers P2RY12 and TMEM119, coinciding with selective loss of neurons in cortical layers II and III [211]. Of note, the effects of meningeal inflammation were evaluated across lesions and normal-appearing cortical areas, and thus the results may be related to lesional changes [211].

Further insights into the role of microglia and cortical damage come from studies exploring the role of the HLA-DR15 haplotype on microglial density and activation state. HLA-DR genes encode the β and α chains of MHC-II molecules, which are expressed by antigen-presenting cells like microglia. The HLA-DR15 haplotype, which contains the -DRB1*15:01 and -DRB5*01:01 alleles, represents the strongest genetic risk factor in Caucasian people with MS, with estimates of its contribution reaching up to 60% of the total hereditary predisposition [146]. Interestingly, *HLA-DRB1* is the most upregulated gene when comparing MS NAGM and control GM [50]. Carriers of the HLA-DR15 allele exhibit higher *HLA-DRB* expression, which correlates with increased HLA-DRA levels, implying that more functional MHC class II molecules are present on the microglia plasma membrane. Increased MHC class II may enhance antigen presentation and subsequent CD4^+^ T cell activation [50]. In a cohort of young people with MS with severe disease, the HLA-DR15 haplotype was associated with higher cortical lesion load and increased CD68 expression levels [231]. A follow-up study further linked this haplotype to reduced TMEM-119 expression and a smaller neuronal area [232]. Overall, these findings suggest that HLA-DRB1*15:01-expressing microglia may contribute to neuronal damage. Stratifying the MS population depending on the HLA-DR15 status may aid in identifying subgroup-specific disease mechanisms.

In conclusion, transcriptional profiling and immunohistochemical analyses strongly indicate that microglia clusters in NAWM are a pathological entity in MS capable of contributing to CNS tissue damage. Although conclusive evidence is still lacking, their apposition to CRYAB-positive oligodendroglia and demyelinated axons as well as their roles in lipid processing implicate oligodendroglia stress and myelin disturbances as potential drivers of nodule formation. Given that nodules occur throughout the brain, while lesions are often located in proximity to blood vessels or cerebrospinal fluid-filled spaces, it is plausible that oligodendrocyte and myelin damage represent a first hit that only culminates in classic demyelinating plaques when additional blood- or cerebrospinal fluid-borne (immune) factors contribute to the pathological processes. In NAGM, microglial nodules have not yet been observed, although animal studies suggest that microglial clustering is an early response to myelin damage in the GM as well [169]. However, the mechanisms underlying microglia-mediated damage in NAGM and NAWM likely differ. In NAGM, transcriptional data and immunohistochemistry studies (see following section, 5.1 on Synaptic pathology within NAGM) point to excessive microglial synaptic pruning as a key factor that could ultimately lead to neuronal damage. Overall, the intricate interplay between microglia and the other CNS cellular components highlights microglia’s central role in the pathogenesis of MS and possibly their functions in tipping the balance between further damage or tissue repair.

## Astrocytes: friends or foes?

Astrocytes play a crucial role in maintaining CNS homeostasis by regulating blood flow, the blood–brain barrier [66], neurotransmitter levels, and ion balance [168]. Beyond these functions, they support to oligodendrocytes and neurons: astrocytes store energy in the form of glycogen, which is metabolized into pyruvate and lactate and transferred via monocarboxylate transporters to oligodendrocytes or myelin, ultimately reaching axons [58]. Astrocytes also transfer mitochondria to damaged neurons [71] and supply lipids necessary for myelination [24] and OPC differentiation [110]. While the responses of astrocytes following demyelination are well studied, their involvement in the early stages of lesion formation or during progression is largely overlooked. Nonetheless, astrocytes are altered in NAWM and NAGM, warranting further investigation into their early pathological contributions.

In both NAGM and NAWM, astrocytes exhibit elevated levels of phosphorylated inositol-requiring enzyme 1 alpha (IRE1α) and spliced X-box binding protein 1 (XBP1), indicating activation of the unfolded-protein response [222]. In the mouse model experimental autoimmune encephalomyelitis (EAE), where MS-like symptoms are induced through autoimmune-mediated destruction of myelin, silencing the IRE1α/XBP1 axis attenuates symptom severity [35]. Interestingly, unlike in NAGM and NAWM, astrocytes within WM lesions do not exhibit activation of the IRE11α/XBP1 axis (although other cell types do) [222], suggesting that this pathway may play an early role in astrocyte-mediated damage. Differential gene expression analysis comparing MS NAGM and control GM identified a significant downregulation of astrocyte-specific genes involved in the astrocyte-neuron lactate shuttle and the glutamate-glutamine cycle [234], which could result in reduced lactate shuttling to neurons and dysregulation of neurotransmitter levels. Such metabolic changes were accompanied by downregulation of connexin 43 (Cx43) [234], a protein coupling astrocytes and oligodendrocytes into a panglial syncytium that contributes to axonal support [200]. At the protein level, Cx43 hemichannels were also selectively lost within cortical layers III–V of NAGM [234]. This layer-specific reduction suggests an underlying cell-autonomous mechanism, rather than one driven by immune signals emanating from the meninges (expected to cause the greatest Cx43 reduction in layer I with a gradual decline toward layer III–V) or from blood vessels (which would uniformly affect all layers). By contrast, a separate study reported elevated expression levels of Cx43 in NAGM compared to control GM, particularly in layers I and II, indicating a gradient of astrogliosis toward the cortical surface [121]. Overexpression of Cx43 induces release of glutamate, ATP, and cytokines into the extracellular milieu [32, 148], possibly exacerbating tissue damage. In line with this hypothesis, pharmacological blockade of Cx43 hemichannels slowed disease progression in the EAE model [190]. Further implicating astrocytes as contributors to disease pathology, an early study reported depletion of β2-adrenergic receptors within astrocytes from both NAWM and WM lesions [43]. These receptors, upon binding to their ligand norepinephrine, regulate key cellular functions, such as secretion of trophic factors, lactate production, and MHC-II expression [42]. As reviewed elsewhere, loss of these receptors could contribute to features of MS pathology, such as myelin damage and axonal degeneration [42].

Astrocytes may also exert beneficial effects within the NAWM and NAGM. A transcriptomic analysis of GFAP-expressing astrocytes isolated from MS NAWM and control WM revealed upregulation of genes related to iron homeostasis and oxidative stress, implying a role in mitigating iron-induced oxidative damage [217]. Additionally, the neurotrophic factor transforming growth factor beta 3 (TGF-β3) was upregulated and the inflammatory mediator cyclooxygenase-2 (COX-2) was downregulated in NAWM astrocytes compared to WM astrocytes from controls, further supporting a protective function [217].

Astrocytes, along with other cell types, contribute to the extracellular matrix (ECM) by secreting various components. During astrogliosis, they actively remodel the ECM through transcriptional programs that enhance the secretion of matricellular proteins, particularly chondroitin sulfate proteoglycans (CSPGs) [127] and hyaluronic acid [28]. A deeper understanding of this process within humans is of particular relevance for developing therapies for MS, given CSPGs inhibit OPC differentiation and hinder remyelination in vivo [91]. An early study found that MS NAWM from spinal nerve roots contains granular aggregates of the CSPGs aggrecan and versican, alongside a reduction in neurocan compared to control WM, suggesting widespread alterations in CSPG composition [184]. However, analysis of the ECM transcriptome in MS NAWM revealed minimal changes, with only the hyaluronan receptor CD44 being significantly upregulated in NAWM compared to control WM [187]. Nonetheless, a recent transcriptomic study identified a subpopulation of people with MS characterized by an increased ECM response that inhibits OPC differentiation, a signature that was maintained across both lesioned and normal-appearing areas [116]. Thus, the evidence regarding ECM alterations in NAWM is still incomplete, potentially due to MS disease heterogeneity.

In summary, the role of astrocytes in normal-appearing MS tissue remains largely underexplored. Current research supports a dual role: some studies indicate that astrocytes exhibit neurotoxic behavior and abnormal crosstalk with oligodendrocytes, while others emphasize their neuroprotective functions. In support of their protective roles, iPSC-derived astrocytes from a patient with a benign course of MS could rescue neuronal damage induced by inflammatory cytokines [92]. Despite these conflicting interpretations, the overall evidence suggests that aberrant astrocyte reactivity and/or dysfunction in MS is more likely a secondary rather than a primary driver of pathology. Nonetheless, considering astrocytes’ significant contributions to processes, such as antigen presentation, regulation of BBB homeostasis, lipid supply for myelin repair, and iron redistribution, the balance between their beneficial and damaging functions conceivably directs the pathologic fate of NAWM toward a full-blown lesion or a silent state, and contributes to determining the course and severity of MS pathology.

## Neurons: just silent actors behind the scenes?

Neurodegenerative processes affecting nearly all neuronal compartments are present from early on in the MS disease course, resulting in the development of premature widespread atrophy. GM pathology may develop independently of WM damage [107] and is potentially driven by mechanisms unrelated to relapse-associated inflammation and demyelination [226] although this remains a subject of ongoing debate. Damage to GM structures possibly plays a central role in SAW, which is hallmarked by symptoms, such as sensory dysfunction, cognitive decline, and fatigue in the absence of clear relapses or MRI activity related to lesions [174]. Thus, a deeper understanding of the pathways leading to neurodegeneration may aid in the development neuron-targeted interventions aimed at halting smoldering MS.

### Synaptic pathology affects NAGM throughout the CNS

While synapse loss is a critical feature of GM lesions in MS [219], it also occurs in normal-appearing cortical regions (Fig. 3C) [79, 84, 213]. Reconstructions of single pyramidal neurons in layers IV–VI of the insular, frontotemporal, and occipital lobes of people with MS showed a marked reduction of spine density (more than 50%) compared to non-neurological controls [84]. Surprisingly, such reduction was comparable to that of lesioned areas. By contrast, axonal density was reduced only in cortical lesions and not in NAGM. Thus, diffuse spine loss in the NAGM is likely a primary phenomenon rather than a secondary consequence of reduced synaptic input due to axonal degeneration. Similarly, both excitatory and inhibitory synapses are selectively reduced in normal-appearing regions of layer VI of the superior frontal cortex in people with MS [79]. In this study, although axonal density was not evaluated, no loss of excitatory or inhibitory neurons was observed, further supporting the existence of synaptic demise in the absence of neuronal death. As cortical layer VI mainly projects to the thalamus, which undergoes early atrophy in MS [6], retrograde trans-synaptic degeneration may have caused the observed synaptic loss. In layer V of the inferior frontal sulcus and superior temporal sulcus of people with MS, non-selective synaptic loss affects both GABAergic and glutamatergic terminals [213]. In these regions, both neuronal density and axon bundle width are reduced in NAGM compared to control GM, but synapse density does not correlate with either of these changes [213]. Interestingly, an inverse correlation between synapse density in NAGM and disease duration suggests that synapse loss may occur in early disease stages or in more aggressive clinical presentations [213].

Synapse loss is not limited to the neocortex, but also extends to the NAGM of the basal nuclei [213], hippocampus [163], cerebellum [2], and spinal cord [158]. In the normal-appearing hippocampus of people with MS, synaptic alterations included a reduction in inhibitory synapses and an increase in the excitatory presynaptic element Homer1 [163]. The loss of inhibitory synapses was accompanied by loss of inhibitory interneurons, suggesting that the reduction in inhibitory synapses might have resulted from interneuron loss [163]. The unexpected increase in glutamatergic presynaptic elements could be attributed to an increase in reactive astrocytes—previously reported to express Homer1 [20]—rather than neuronal synapses. Additionally, synaptic alterations correlated with increased levels of the complement component C1q, which targets synapses for removal [45], suggesting that complement-licensing may mediate aberrant synaptic pruning in MS [163]. In the cerebellar dentate nucleus of people with MS, synaptic density and the number of synapses per neuron were decreased to the same extent in demyelinated and non-demyelinated areas when compared to tissue from non-neurological controls [2]. Ultrastructural analyses revealed phagolysosomes containing degraded synaptic vesicles localized within neuronal dendrites, providing evidence of a neuron-autonomous mechanism for synapse degradation [2]. In the spinal cord, synaptophysin levels and synaptic button area were reduced in both lesioned and normal-appearing areas of people with MS when compared to control tissue, with more severe pathology in lesioned areas [158].

The similar extent of synapse loss in both GM lesions and NAGM strongly suggests that, at least in some CNS regions, synapse elimination is an early event in MS pathology occurring independently of demyelination and potentially preceding it [220]. Moreover, the non-selective nature of synapse loss, affecting both excitatory and inhibitory terminals, points to a broad mechanism of synapse removal rather than a targeted one. Current evidence highlights two processes as the perpetrators of synapse loss: microglia/complement-mediated pruning and neuron-autonomous degradation. Potential triggers for these processes include (1) de-afferentiation due to axonal transection or lack of afferent input, (2) retrograde trans-synaptic degeneration, and (3) neuronal stress due to excessive cytokine signaling from inflamed meninges or demyelination. The relative contribution of these mechanisms may vary across different regions and disease states, contributing to the complexity of synaptic pathology in MS. Future research is essential to unravel the precise mechanisms behind synapse loss and determine whether it contributes to neurodegeneration or serves as a protective response against potentially harmful events, such as glutamate excitotoxicity and network overload.

### Axonal initial segment lengthening and axonal loss

The axon initial segment (AIS) is an unmyelinated region of the axon rich in Na^+^ and K^+^ channels. Located adjacent to the soma, the AIS plays a crucial role in maintaining neuronal polarity and initiating action potentials. Features of the AIS, such as its length, influence neuronal excitability and allow for a fine-tuned regulation of neuronal circuits [99]. Studies using animal models of MS, such as EAE or cuprizone-fed mice have revealed AIS dysregulation associated with demyelination, thus poising investigations in MS tissue [36, 68]. Cortical MS lesions present increased distance between the soma and AIS and increased AIS length [180]. NAGM Purkinje cells also show a longer soma-AIS gap compared to control tissue, suggesting that AIS alterations might be an early event in the cascade of events culminating in lesion formation [180]. According to computational models, the heightened AIS-soma distance could lower the action potential threshold and could thus reflect a compensatory mechanism aiming to restore neuronal properties, similar to how the reorganization of axonal Na^+^ channels upon demyelination allows action potential propagation [180]**.**

Axonal loss occurs in GM lesions [49, 198] as well as in NAGM [97, 213] and NAWM [104] (Fig. 3D). In frontal NAGM areas, axonal loss may reach up to 33% compared to control tissue [97]. In frontal and temporal NAGM regions, the width of axonal bundles is reduced by 37% compared to control cortices [213]. NAGM axonal loss correlates with reduced integrity of connected NAWM tracts, as reported by MRI proxies like axial diffusivity and mean diffusivity [95]. This suggests that a tract-specific “dying back” mechanism may partially contribute to axonal loss within NAGM. In NAWM, axonal degeneration was observed throughout the whole hemispheric section although it increased in the vicinity of WM lesions [104]. No correlation was found between NAWM axonal injury and WM lesion load or cortical demyelination, indicating that these features may occur through at least partly independent mechanisms [104].

The extent of axonal degeneration is likely variable across different brain regions and disease stages. For example, some studies report no significant reduction in tangentially oriented cortical fibers in NAGM areas of the insular and frontotemporal lobes compared to controls [84] while extensive NAGM axonal loss has been reported in other NAGM areas. Additionally, NAWM axonal loss is more pronounced in patients with long disease duration [104]. Axonal degeneration is likely also influenced by axonal diameter. Axons with the smallest diameters may be particularly vulnerable to degeneration in MS [147, 191], possibly due to their lower energetic reserves or greater susceptibility to ROS and other toxic mediators. Supporting energy dysfunction in MS axons, normal-appearing optic nerve axons from people with MS show an increased mitochondrial density [205], possibly reflecting heightened energy demands due to nodal disturbances.

### Selective neuronal loss within NAGM

Whether the previously described neuronal alterations are accompanied by neuronal loss is a subject of debate as evidence of neuronal degeneration within NAGM is somewhat conflicting. In a seminal article, Magliozzi et al*.*, demonstrated significant neuronal loss in the layers I–IV of normal-appearing cortex only in MS cases presenting B cell–enriched follicles in the overlying meninges [118]. Although other studies have not found neuronal loss within NAGM [97, 219], the lack of significant differences may be due to a plethora of factors, including that analyses were often conducted across all neuronal layers or without considering the presence or absence of meningeal lymphoid follicle-like aggregates. Nonetheless, some of these studies reported subtle alterations hinting at neuronal stress in NAGM, such as increased circularity (indicative of cytoskeleton breakdown) or reduced somata volume [97, 219].

Other studies find a selective loss of certain neuronal populations in MS NAGM [176, 235]. A single-nucleus RNA sequencing study revealed selective loss of excitatory CUX2-expressing neurons in layers II/III often underlying meningeal inflammation. Although CUX2^+^-neurons loss was observed only in demyelinated regions, stress markers (such as prolyl isomerase cyclophilin A, involved in the ROS response [46]) were also observed within CUX2^+^-neurons in NAGM, suggesting a gradient of pathology [176]. Additional studies find markers of neuronal stress within normal-appearing cortical areas [130], such as mislocalization of the heterogeneous nuclear ribonucleoprotein A1 (hnRNP A1). hnRNP A1 mislocalization was detected within NAGM neurons in a subgroup of people with MS with slowly expanding lesions, and was associated to neuronal degeneration and brain atrophy [130]. Although the precise mechanisms of hnRNP A1-mediated damage were not elucidated, in vitro evidence demonstrates that hnRNP A1 dysfunction exacerbates oxidative stress and induces stress granules formation [37] and impairs energy production [153]. CUX2^+^-neuronal loss however was not replicated in another study using immunohistochemistry techniques [235]. In contrast, a significant reduction in parvalbumin (PV)-expressing interneurons was observed in both GM lesions and NAGM [235]. Follow-up experiments in which the demyelinating agent lysophosphatidylcholine was applied to mice cortices confirmed that PV^+^-inhibitory interneurons are more vulnerable to myelin damage than excitatory neurons like the CUX2^+^ population [235]. This increased susceptibility may be attributed to the fact that PV^+^ interneurons are among the most heavily myelinated neuronal subtypes [23]. Damage to the myelin sheath of PV^+^ interneurons could impair the metabolic support provided by myelin, while also limiting access to the nutrient-rich extracellular space [175], and could thus underlie PV^+^ loss in GM lesions.

In the spinal cord, modest neuronal loss occurs within NAGM [63, 158]. The subtle-to-mild loss of neurons observed, often accompanied by a pronounced axonal loss in the corticospinal tracts, indicates a remarkable resilience of spinal cord neurons to trans-neuronal degeneration.

Damage affects all neuronal compartments in the NAGM of individuals with MS, resulting in diffuse synaptic dysfunction, axonal transection and loss, and neuronal demise. However, to date, the temporal sequence and triggers of these neurodegenerative events remain largely elusive although potential contributors include: (a) secretion of neurotoxic factors by meningeal B cell infiltrates, (b) retrograde or anterograde degeneration resulting from demyelination or axonal transection in lesions affecting connected regions, (c) microglia-mediated synaptic pruning, or (d) selective vulnerability of certain neuronal populations due to factors like higher metabolic demands and thus higher susceptibility to demyelination. The relation between neurodegenerative processes and cortical lesion formation is uncertain. Is GM myelin loss a consequence of neuronal damage, or are these two independent events? Given the potential contribution of both focal and diffuse GM pathology to MS symptoms, such as chronic fatigue and cognitive decline, further investigation into the underlying pathophysiological mechanisms remains a critical challenge in the field.

## Lymphocytes: driving NAWM progression into lesions

Traditionally, MS has been regarded as a T cell mediated autoimmune disease, with autoreactive T cells playing a central role in lesion formation. Aside from the MHC genetic region, the majority of MS risk alleles are associated with T cell functions [80]. Histological studies have revealed a twofold increase in T cell density in NAWM compared to the WM of healthy controls [205]. In NAWM, T cells are found both in the parenchyma and in perivascular spaces [104, 205], and form perivascular cuffs particularly in patients with progressive disease [104]. NAWM T cell density has been negatively correlated with myelin density and paranodal and juxtaparanodal abnormalities [205], suggesting that T cells may exacerbate pre-existing tissue damage for example via the secretion of cytotoxic cytokines. Supporting this, the CD8^+^ subset of T cells has been identified in perivascular spaces of NAWM [142] as well as in WM lesions [115, 142]. In cuprizone-fed mice, demyelination has been associated with presentation of a neuron-specific antigen to specific CD8^+^ cells, leading to axonal injury [38]. In this model, CD8^+^ T cells activated by demyelination were also found to infiltrate not lesional tissue [38], suggesting that MS lesion-associated CD8^+^ T cells could infiltrate into adjacent normal-appearing tissue and contribute to at least some of the alterations found within NAWM.

The success of B cell targeted therapies over the past two decades has underscored the role of B cells as another key player in the pathogenesis of MS. B cell aggregates are found within meninges and parenchyma of the CNS and associate with an unfavorable prognosis in MS [57]. Selective enrichment of B cells that have maturated into antibody-secreting cells in NAWM and WM lesions compared to peripheral blood has been reported by flow cytometry [17]. However, according to IHC, NAMW did not present higher numbers of antibody-secreting cells compared to peripheral blood although reactive areas and active and mixed lesions did [17]. Total IgA and IgM gene expression levels were significantly increased in MS NAWM compared to control WM [17]. IgG levels were similar between MS NAWM and control WM, although a trend toward higher levels in MS NAWM was observed [17].

Antibodies likely play a direct role in myelin and axonal destruction and could explain to some extent pathology seen in normal-appearing and lesioned regions. Evidence supporting this notion comes from studies in which antibodies from plasmablast clones of people with MS induced myelin damage, astrocyte activation, and complement deposition when injected into the brains of mice [16, 108, 150]. Autoantibodies against the axonal proteins neurofascin-155/186, which contribute to the axo-glial interactions at the paranodes, were particularly prevalent in patients with late-stage progressive MS [125]. When these antibodies were transferred into EAE mice, they led to increased axonal damage and exacerbated disease severity [125]. In another study, cerebrospinal fluid IgG specifically derived from patients with primary progressive MS induced demyelinating lesions and reactive gliosis when injected in the subarachnoid space of mice [224]. Interestingly, no pathogenic effect was observed with IgG derived from patients with secondary progressive MS or relapsing remitting MS [224]. An early study classified MS lesions into four types, with only one (pattern II) showing involvement of antibodies and complement in demyelination, suggesting distinct disease mechanisms among patients [112]. While pattern II is mainly found in relapsing–remitting MS, it also occurs in primary progressive MS [196]. Thus, the presence of pathogenic IgG exclusively in primary progressive MS CSF is unexpected and warrants further investigation to clarify the underlying mechanisms. Despite this discrepancy, the available evidence collectively supports that B cell–derived antibodies contribute to tissue damage in NAWM in at least a subset of people with MS.

## Pathological relevance of normal-appearing tissue alterations

### Normal-appearing tissue alterations possibly contribute to smoldering clinical disability

The use of potent disease-modifying treatments effectively suppresses immune-driven relapses, yet only minimally impacts MS progression, revealing PIRA as the main driver of disability accrual through the disease course [111, 161, 201], even from the early stages of disease [86, 87]. Several pathobiological mechanisms, both upstream and downstream of lesion formation, may underlie PIRA, including slowly expanding lesions, leptomeningeal inflammation, chronic energy deficits and oxidative insults, and widespread microglial reactivity. For a more comprehensive overview, we refer the reader to excellent reviews on this topic [22, 64]. A relatively unexplored hypothesis is that the accumulation of pathological alterations in NAWM and NAGM is a pathological substrate for PIRA.

Evaluation of microglial state using positron emission tomography (PET) imaging with radioligands that bind to the 18 kDa translocator protein (TSPO) found that people with MS with heightened microglial density in NAWM were more likely to experience clinical progression in the absence of new relapses or new MS lesions [189]. Additionally, microglial density according to TSPO-PET imaging [183] and brain microstructure [67] correlated with cognitive fatigue. Interestingly, increased microglial density in NAWM correlates with surrogate measures of microstructural myelin damage using diffusion tensor imaging, and both microglial density and myelin damage were linked to clinical progression using the Expanded Disability Status Scale (EDSS) [14]. Currently available in vivo evidence addressing whether microstructural myelin damage is related to disability accrual is however conflicting, with some studies reporting positive correlations, while others do not (extensively reviewed elsewhere [94]). In these studies, accumulation of disability is often evaluated using EDSS, a widely used clinical scale of motor performance in the assessment of PIRA. However, SAW often does not present as motor deficits measured by EDSS, but rather as subtle subjective complains that are only captured by other clinical tests assessing general well-being, fatigue, or the cognitive domain [174].

To date, PIRA and SAW remain a major unmet clinical need. Immunomodulating therapies that reduce relapse-associated worsening minimally impact PIRA although a second-generation BTK inhibitor has recently shown promise, meeting its endpoint in a phase III clinical trial [237]. As therapeutic implications will arise from a better understanding of the pathobiology underlying SAW, it is necessary to further assess whether NAWM and NAGM alterations underlie progressive clinical worsening by implementing tests better tailored at assessing subtle complaints.

### NAWM and NAGM alterations: an integrated perspective

**Fig. 4:**
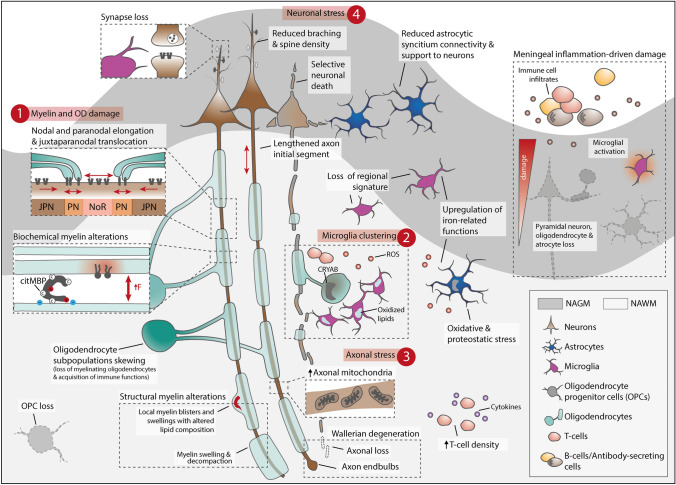
Overview of widespread abnormalities in normal-appearing gray matter (NAGM) and normal-appearing white matter (NAWM) that may synergistically contribute to disability accumulation. (1) Metabolic and functional dysregulation of oligodendrocyte progenitor cells, oligodendrocytes, and myelin may result from cell-intrinsic mechanisms, from damage from nearby lesions or diffusely abnormal white matter, or the overall proinflammatory environment within the MS brain. Myelin segments, possibly with aberrant lipid profile and/or excessive MBP citrullination, may have a reduced attachment between the lamella, and therefore may decompact or present with structural abnormalities, such as myelin blisters or myelinosomes. Oligodendrocyte stress and myelin alterations could thus favor demyelination. (2) Microglial nodules form around partially demyelinated axons and actively phagocytose myelin debris. While some nodules likely resolve, others may persist and evolve into chronic microglial activation, adopting a proinflammatory phenotype that impairs the repair process. Myelin damage may be further exacerbated by microglia- and lymphocyte-derived factors, such as proinflammatory cytokines and reactive oxygen species. In the context of a persistently proinflammatory environment, intrinsic oligodendrocyte dysfunction and secondary microglial responses may converge to disrupt the myelin sheath. This cascade can initiate a ripple effect leading to widespread demyelination and lesion formation, potentially amplified by lymphocyte infiltration through blood vessels. (3) Axonal energy deficiency**,** whether caused by subtle myelin damage or overt demyelination, may elicit adaptive neuronal responses, including an increased number of axonal mitochondria. However, the failure of these compensatory mechanisms—alongside retrograde and Wallerian degeneration originating from distal lesions—ultimately contributes to severe axonal damage. (4) In the NAGM, neuronal stress caused by axonal damage in the NAWM, nearby or distant lesions, and meningeal inflammation can result in subtle signs of neuronal injury—such as synaptic puncta retraction—and may ultimately lead to neuronal loss, particularly in subpopulations with the greatest energetic demands. *OPCs*  oligodendrocyte progenitor cells, *ROS* reactive oxygen species, *CRYAB*   α-crystallin B, *JPN*  juxtaparanodes, *PN*   paranodes, *NoR*  nodes of Ranvier, *citMBP*  citrullinated myelin basic protein

Oligodendrocytes, microglia, astrocytes, and neurons exhibit prominent alterations in both NAWM and NAGM (Fig. 4). Among the studies reporting alterations in MS NAWM, those related to myelin alterations remarkably outnumber any other. Structurally and compositionally, myelin in MS NAWM exhibits diverse features that are predicted to reduce its resistance and increase capacitance, and thus likely slow or halt axonal transmission. Certain structural changes, such as myelin blisters and myelinosomes, may also disrupt axo-myelinic communication, thereby compromising the metabolic support that myelin provides to their enwrapped axons that are—other than at the nodes of Ranvier—isolated from other sources of metabolites [139]. Although myelin is essential for axonal integrity, damaged myelin poses a greater threat to axonal health than its complete absence [175], showcasing that “bad myelin is worse than no myelin” [138, 139].

Specific structural myelin disturbances, including nodal and paranodal disruptions and increased g-ratio within normal-appearing optic nerves, correlate with the extent of microglial clustering in the surrounding tissue, suggesting a link between microglia nodules and myelin abnormalities [205]. Lymphocytes in the vicinity of microglial nodules contribute to an intensified proinflammatory environment, potentially tipping the balance in NAWM toward lesion formation [206]. Further supporting the role of microglia nodules in early myelin damage, nodules are often juxtaposed to demyelinated or degenerating axons [182, 205] and oligodendrocytes expressing chaperones involved in stress response [210]. Addressing the chicken and egg story however remains challenging as often human post-mortem research provides evidence for both directions of causality. Of note, in perilesional WM, oligodendrocyte density does not negatively correlate with microglial density [93, 210], suggesting that microglia may not directly mediate oligodendrocyte loss and hinting at intrinsic, cell-autonomous, oligodendrocyte dysregulation. Further supporting this, iPSCs from people with primary progressive MS exhibit impaired differentiation into mature oligodendrocytes [39]; and a genetic study identified two loci associated with MS risk that impact RNA polymerase activity in oligodendrocytes [51]. These findings suggest that myelin damage in MS may not solely spread centripetally (from the myelin processes to the oligodendrocyte soma), as convention holds. Instead, intrinsically dysregulated processes within oligodendrocytes could additionally contribute to myelin damage that propagates centrifugally (from the cell body to the myelin processes), potentially playing a role in disease progression. Consistent with this, targeted damage to the oligodendrocyte cell body causes myelin degeneration prior to soma loss in vivo [31].

Further underscoring the significance of intrinsic glial dysfunction in NAWM and challenging the traditional myelin-centric perspective of MS pathology, single-cell analysis of NAWM from a large cohort of people with MS revealed distinct patient subgroups defined by unique transcriptional profiles in glial cells [116]. Notably, the transcriptional signature that identified each patient subgroup was maintained regardless whether the cells were located within WM lesions or in NAWM, indicating that the signatures were largely independent of the lesion environment and were instead patient-specific. The transcriptional signatures identified were: (1) a cross-glial stress response involving increased protein folding, molecular chaperones, and ubiquitination; (2) a cross-glial stress response associated with DNA damage; (3) an inhibitory extracellular matrix response linked to oligodendroglia degeneration; and (4) an immune-associated oligodendrocyte response coupled with impaired oligodendrocyte maturation [116]. A reparative astrocyte response was also observed, with varying degrees of extent between patients and subgroups [116].

In contrast to NAWM, literature about NAGM is dominated by studies about neuronal alterations, ranging from synaptic changes as severe as those seen in lesions to subtle losses of certain neuronal subpopulations. A key factor mediating neurodegeneration could be pro-inflammatory microglia. Microglia-mediated neuronal damage may occur via secreted factors with neurotoxic effects [134], but also through direct contact (e.g., at synapses [199] or the AIS [36]). Compromised myelin function might also contribute to neuronal vulnerability. Despite the absence of structural abnormalities, NAGM myelin shows an altered lipid profile [147, 193]. Recent findings indicate that myelin lipids can act as an energy source when glucose is limited [5, 164]. Therefore, albeit hypothetical, the altered NAGM myelin lipid composition could impair metabolic support to neurons. As fast-spiking neurons are particularly sensitive to metabolic disruptions, even slight impairments in myelin metabolic support could have far-reaching consequences for neuronal health.

Although the exact mechanisms underlying NAGM and NAWM abnormalities remain unclear, they likely result from a combination of both intrinsic cellular dysregulation and cellular responses to local or distant damage in lesions or in diffusely abnormal white matter areas through processes like anterograde or retrograde degeneration. Dysregulated processes across cell types converge on common mechanisms, particularly oxidative stress, lipid imbalance, and the acquisition of aberrant proinflammatory roles—likely reflecting the overall proinflammatory environment of the MS brain. Understanding how CNS cells transition from homeostatic to pathogenic states within normal-appearing regions would aid in elucidating the processes leading to both lesion formation, smoldering progression, and may uncover novel therapeutic targets.

### Do normal-appearing tissue changes occur early in the disease, or result from chronic CNS damage?

A particularly difficult question to answer is whether the cellular and biochemical alterations within normal-appearing tissue in MS are present from disease onset or simply represent a consequence of long-standing damage of neighboring tissue, such as through Wallerian degeneration. The evidence described in this review concerns post-mortem histopathological findings mainly derived from late progressive MS cases with high pathological burden. However, studies in living patients support that alterations of myelin, axons, and microglia are already present in early disease stages in MS although these changes tend to become more pronounced over time, likely due to the accumulation of disease-related damage and age-related factors (see Box 2). Advanced quantitative multi-shell imaging techniques, such as neurite orientation dispersion and density imaging [162] or composite hindered and restricted model of diffusion [44], indicate widespread neurite damage in both NAGM and NAWM areas even in relapsing–remitting phases of the disease (extensively reviewed elsewhere [27]). Moreover, serum neurofilament light chain levels—a marker of neurodegeneration—increase as early as six years before clinical MS onset, supporting that subtle tissue damage may precede lesion formation although it cannot be excluded that such an increase is driven by preclinical, silent lesions [15]. Early diffuse myelin damage is also found in both NAWM and NAGM since early disease stages using multi-shell and myelin imaging techniques though it is less prominent than axonal damage [162]. Interestingly, early microstructural myelin damage indicated by diffusion tensor imaging metrics positively correlates with microglial density detected through TSPO-PET [14], supporting an interplay between myelin damage and microglial reactivity. Further supporting an early role of microglial alterations, higher TSPO binding in patients with clinically isolated syndrome predicts clinical conversion to MS [62]. While TSPO ligands offer a means to measure microglial and astrocytic density and activation [145] valuable for mapping the general landscape of the brain, TSPO-based techniques fall short in capturing cell type pathology details, such as those revealed by single-cell RNA sequencing. Moreover, imaging techniques are limited in their ability to assess cell types other than microglia and astrocytes, such as oligodendrocytes. Developing more specific PET tracers that can bind to different CNS cell types is a critical avenue for advancement, as they could provide valuable insights into these cells in patients in the early stages of MS.

Box 2 Aging and MS: a two-way street Aging is one of the most significant factors influencing disease progression, with the onset of progression more closely tied to age than to the duration or presence of pre-progression symptoms [202]. Here, we describe the evidence suggesting that aging may exacerbate multiple sclerosis (MS)-related degeneration in normal-appearing white and gray matter (NAWM/NAGM).

During aging, cellular and molecular changes accumulate progressively, including senescence, mitochondrial dysfunction, defective autophagy and mitophagy, inflammasome activation, dysregulation of the ubiquitin–proteasome system, and chronic activation of the DNA damage response (extensively reviewed in Ref. [55]). These changes impair immune system function, a process further aggravated by increased exposure to microbial molecules stemming from heightened gastrointestinal permeability [194]. Collectively, these drive “inflammaging”, a state of chronic, low-grade inflammation [54]. In the CNS, aging microglia adopt a “primed” phenotype, characterized by heightened reactivity and excessive secretion of inflammatory cytokines, possibly partly driven by peripheral low-grade inflammation [225]. Neuronal loss and reduced neuron–microglial interactions, such as those mediated by CD200 [56], also occur concomitant with aging and possibly contribute to microglial hyperreactivity. Despite increased reactivity, aged microglia display reduced phagocytic capacity [90], which, alongside age-related myelin abnormalities [156, 172] and degeneration [74], surpasses their clearance capacity [25, 123, 171, 172]. This overload leads to the accumulation of insoluble lipid and protein aggregates within microglia, triggering inflammasome activation [25] and intensifying the pro-inflammatory response [123]. This dual dysfunction—hyperreactivity coupled with inefficient phagocytosis—creates a microglial population prone to overreacting to damage by secreting excessive inflammatory cytokines, while being less effective at clearing myelin debris, possibly compounding existing pathology. Notably, microglial depletion in rodents induces myelin outfoldings, followed by myelin loss, and both phenotypes are aggravated with aging [128]. Thus, microglial dysfunction may also contribute to age-associated myelin damage. Additionally, transcriptional data indicate enhanced antigen presentation and phagocytosis within aged oligodendrocytes [229].

In MS, where myelin integrity is already compromised and microglia is already highly reactive, it is likely that these age-dependent mechanisms exacerbate pathology. Many of the events occurring with aging further aggravate in MS, such as paranodal disruption, possibly contributing to axonal conduction defects [59], or CD200 loss, contributing to microglial hyperreactivity [207]. Supporting this, glial cells within NAWM present an accelerated epigenetic aging according to DNA methylation profile when compared with that of healthy individuals [103]. Although aging-related degeneration and MS pathology are distinct processes, they likely interact synergistically to worsen disease outcomes. Functions that are already impaired by MS become further compromised with aging, creating a feedback loop of escalating dysfunction. Therefore, understanding the interplay between MS and aging is essential for developing targeted interventions to address the combined impact of these drivers of CNS damage. In MS, where myelin integrity is already compromised and microglia is already highly reactive, it is likely that these age-dependent mechanisms exacerbate pathology. Many of the events occurring with aging further aggravate in MS, such as paranodal disruption, possibly contributing to axonal conduction defects [59], or CD200 loss, contributing to microglial hyperreactivity [207]. Supporting this, glial cells within NAWM present an accelerated epigenetic aging according to DNA methylation profile when compared with that of healthy individuals. Although aging-related degeneration and MS pathology are distinct processes, they likely interact synergistically to worsen disease outcomes. Functions that are already impaired by MS become further compromised with aging, creating a feedback loop of escalating dysfunction. Therefore, understanding the interplay between MS and aging is essential for developing targeted interventions to address the combined impact of these drivers of CNS damage.

### Concluding remarks

This review provides a comprehensive description of the changes affecting normal-appearing brain tissue in MS. While the alterations in NAGM and NAWM differ qualitatively, several perpetrators are at play in both settings: myelin lipid dysregulation, microglial dysfunction, axonal loss, and energy deficits. The demands and the pathological features of WM and GM are however distinct, suggesting that damage in these compartments may occur through largely independent mechanisms. At the same time, NAWM and NAGM should not be viewed as entirely separate, as they are functionally connected. Finally, it is important to acknowledge that possibly not all normal-appearing tissue may be equally affected, and that localized “hotspots” of alterations exist (i.e., microglia nodules).

Based on the evidence discussed in this review, we propose that normal-appearing tissue in MS represents a clinically relevant, pathological entity distinct from demyelinating lesions. Alterations in normal-appearing tissue may not only give rise to overt lesions, but also underlie disability accumulation from the earliest stages of disease, which manifest once repair mechanisms are exhausted. Therefore, we fully align with the emerging view taking distance from the traditional “lesion-centric” paradigm of MS [64, 102]. A wider understanding of the pathophysiology of the “normal-appearing” MS tissue not only provides a more complete picture of disease progression, but also emphasizes the need for therapeutic approaches targeting such diffuse pre-lesional changes to slow or halt disability accumulation in MS.

## Methods

We conducted a literature search using PubMed, focusing on the terms “normal appearing white matter,” “non lesioned white matter,” “normal appearing grey matter”, and “non lesioned grey matter”. The search covered studies published up to March 2025, and primarily targeted articles published within the last decade, although earlier studies were also included if deemed relevant. Of note, the search was not systematic; in many cases, additional articles were identified through references cited in the initially retrieved publications.

## References

[1] Absinta M, Cortese ICM, Vuolo L, Nair G, de Alwis MP, Ohayon J et al (2017) Leptomeningeal gadolinium enhancement across the spectrum of chronic neuroinflammatory diseases. Neurology 88:1439–1444. 10.1212/WNL.0000000000003820 28283598 10.1212/WNL.0000000000003820 PMC5386437

[2] Albert M, Barrantes-Freer A, Lohrberg M, Antel JP, Prineas JW, Palkovits M et al (2017) Synaptic pathology in the cerebellar dentate nucleus in chronic multiple sclerosis. Brain Pathol 27:737–747. 10.1111/bpa.12450 27706868 10.1111/bpa.12450 PMC8028945

[3] Arancibia-Carcamo IL, Attwell D (2014) The node of ranvier in CNS pathology. Acta Neuropathol 128:161–175. 10.1007/s00401-014-1305-z 24913350 10.1007/s00401-014-1305-z PMC4102831

[4] Arancibia-Cárcamo IL, Ford MC, Cossell L, Ishida K, Tohyama K, Attwell D (2017) Node of ranvier length as a potential regulator of myelinated axon conduction speed. Elife 6:e23329. 10.7554/eLife.23329 28130923 10.7554/eLife.23329 PMC5313058

[5] Asadollahi E, Trevisiol A, Saab AS, Looser ZJ, Dibaj P, Ebrahimi R et al (2024) Oligodendroglial fatty acid metabolism as a central nervous system energy reserve. Nat Neurosci 27:1934–1944. 10.1038/s41593-024-01749-6 39251890 10.1038/s41593-024-01749-6 PMC11452346

[6] Azevedo CJ, Cen SY, Khadka S, Liu S, Kornak J, Shi Y et al (2018) Thalamic atrophy in multiple sclerosis: a magnetic resonance imaging marker of neurodegeneration throughout disease. Ann Neurol 83:223–234. 10.1002/ana.25150 29328531 10.1002/ana.25150 PMC6317847

[7] Baaklini CS, Ho MFS, Lange T, Hammond BP, Panda SP, Zirngibl M et al (2023) Microglia promote remyelination independent of their role in clearing myelin debris. Cell Rep 42:113574. 10.1016/j.celrep.2023.113574 38100356 10.1016/j.celrep.2023.113574

[8] Baalman K, Marin MA, Ho TS-Y, Godoy M, Cherian L, Robertson C et al (2015) Axon initial segment-associated microglia. J Neurosci 35:2283–2292. 10.1523/JNEUROSCI.3751-14.2015 25653382 10.1523/JNEUROSCI.3751-14.2015 PMC4315845

[9] Bach D, Epand RF, Epand RM, Miller IR, Wachtel E (2009) The oxidized form of cholesterol 3β-hydroxy-5-oxo-5,6-secocholestan-6-al induces structural and thermotropic changes in phospholipid membranes. Chem Phys Lipids 161:95–102. 10.1016/j.chemphyslip.2009.07.006 19651115 10.1016/j.chemphyslip.2009.07.006

[10] Bacmeister CM, Barr HJ, McClain CR, Thornton MA, Nettles D, Welle CG et al (2020) Motor learning promotes remyelination via new and surviving oligodendrocytes. Nat Neurosci 23:819–831. 10.1038/s41593-020-0637-3 32424285 10.1038/s41593-020-0637-3 PMC7329620

[11] Basilico B, Ferrucci L, Ratano P, Golia MT, Grimaldi A, Rosito M et al (2022) Microglia control glutamatergic synapses in the adult mouse hippocampus. Glia 70:173–195. 10.1002/glia.24101 34661306 10.1002/glia.24101 PMC9297980

[12] Battefeld A, Klooster J, Kole MHP (2016) Myelinating satellite oligodendrocytes are integrated in a glial syncytium constraining neuronal high-frequency activity. Nat Commun 7:11298. 10.1038/ncomms11298 27161034 10.1038/ncomms11298 PMC4866043

[13] Bernier L-P, Bohlen CJ, York EM, Choi HB, Kamyabi A, Dissing-Olesen L et al (2019) Nanoscale surveillance of the brain by microglia via cAMP-regulated filopodia. Cell Rep 27:2895-2908.e4. 10.1016/j.celrep.2019.05.010 31167136 10.1016/j.celrep.2019.05.010

[14] Bezukladova S, Tuisku J, Matilainen M, Vuorimaa A, Nylund M, Smith S et al (2020) Insights into disseminated MS brain pathology with multimodal diffusion tensor and PET imaging. Neurol Neuroimmunol Neuroinflamm 7:e691. 10.1212/NXI.0000000000000691 32123046 10.1212/NXI.0000000000000691 PMC7136049

[15] Bjornevik K, Munger KL, Cortese M, Barro C, Healy BC, Niebuhr DW et al (2020) Serum neurofilament light chain levels in patients with presymptomatic multiple sclerosis. JAMA Neurol 77:58. 10.1001/jamaneurol.2019.3238 31515562 10.1001/jamaneurol.2019.3238 PMC6745051

[16] Blauth K, Soltys J, Matschulat A, Reiter CR, Ritchie A, Baird NL et al (2015) Antibodies produced by clonally expanded plasma cells in multiple sclerosis cerebrospinal fluid cause demyelination of spinal cord explants. Acta Neuropathol 130:765–781. 10.1007/s00401-015-1500-6 26511623 10.1007/s00401-015-1500-6 PMC4655138

[17] Bogers L, Engelenburg HJ, Janssen M, Unger P-PA, Melief M-J, Wierenga-Wolf AF et al (2023) Selective emergence of antibody-secreting cells in the multiple sclerosis brain. EBioMedicine 89:104465. 10.1016/j.ebiom.2023.104465 36796230 10.1016/j.ebiom.2023.104465 PMC9958261

[18] Bongarzone ER, Pasquini JM, Soto EF (1995) Oxidative damage to proteins and lipids of CNS myelin produced by in vitro generated reactive oxygen species. J Neurosci Res 41:213–221. 10.1002/jnr.490410209 7650757 10.1002/jnr.490410209

[19] Bsibsi M, Holtman IR, Gerritsen WH, Eggen BJL, Boddeke E, Valk PVD et al (2013) Alpha-B-crystallin induces an immune-regulatory and antiviral microglial response in preactive multiple sclerosis lesions. J Neuropathol Exp Neurol 72:970–979. 10.1097/NEN.0b013e3182a776bf 24042199 10.1097/NEN.0b013e3182a776bf

[20] Buscemi L, Ginet V, Lopatar J, Montana V, Pucci L, Spagnuolo P et al (2017) Homer1 scaffold proteins govern Ca2+ dynamics in normal and reactive astrocytes. Cereb Cortex 27:2365–2384. 10.1093/cercor/bhw078 27075036 10.1093/cercor/bhw078 PMC5963825

[21] Cairns J, Vavasour IM, Traboulsee A, Carruthers R, Kolind SH, Li DKB et al (2022) Diffusely abnormal white matter in multiple sclerosis. J Neuroimaging 32:5–16. 10.1111/jon.12945 34752664 10.1111/jon.12945

[22] Calabrese M, Preziosa P, Scalfari A, Colato E, Marastoni D, Absinta M et al (2024) Determinants and biomarkers of progression independent of relapses in multiple sclerosis. Ann Neurol 96:1–20. 10.1002/ana.26913 38568026 10.1002/ana.26913

[23] Call CL, Bergles DE (2021) Cortical neurons exhibit diverse myelination patterns that scale between mouse brain regions and regenerate after demyelination. Nat Commun 12:4767. 10.1038/s41467-021-25035-2 34362912 10.1038/s41467-021-25035-2 PMC8346564

[24] Camargo N, Goudriaan A, van Deijk A-LF, Otte WM, Brouwers JF, Lodder H et al (2017) Oligodendroglial myelination requires astrocyte-derived lipids. PLoS Biol 15:e1002605. 10.1371/journal.pbio.1002605 28549068 10.1371/journal.pbio.1002605 PMC5446120

[25] Cantuti-Castelvetri L, Fitzner D, Bosch-Queralt M, Weil M-T, Su M, Sen P et al (2018) Defective cholesterol clearance limits remyelination in the aged central nervous system. Science 359:684–688. 10.1126/science.aan4183 29301957 10.1126/science.aan4183

[26] Caprariello AV, Rogers JA, Morgan ML, Hoghooghi V, Plemel JR, Koebel A et al (2018) Biochemically altered myelin triggers autoimmune demyelination. Proc Natl Acad Sci U S A 115:5528–5533. 10.1073/pnas.1721115115 29728463 10.1073/pnas.1721115115 PMC6003499

[27] Caranova M, Soares JF, Batista S, Castelo-Branco M, Duarte JV (2023) A systematic review of microstructural abnormalities in multiple sclerosis detected with NODDI and DTI models of diffusion-weighted magnetic resonance imaging. Magn Reson Imaging 104:61–71. 10.1016/j.mri.2023.09.010 37775062 10.1016/j.mri.2023.09.010

[28] Cargill R, Kohama SG, Struve J, Su W, Banine F, Witkowski E et al (2012) Astrocytes in aged nonhuman primate brain gray matter synthesize excess hyaluronan. Neurobiol Aging 33:830.e13–24. 10.1016/j.neurobiolaging.2011.07.006 21872361 10.1016/j.neurobiolaging.2011.07.006 PMC3227765

[29] Castegna A, Lauderback CM, Mohmmad-Abdul H, Butterfield DA (2004) Modulation of phospholipid asymmetry in synaptosomal membranes by the lipid peroxidation products, 4-hydroxynonenal and acrolein: implications for Alzheimer’s disease. Brain Res 1004:193–197. 10.1016/j.brainres.2004.01.036 15033435 10.1016/j.brainres.2004.01.036

[30] Cerdán Cerdá A, Toschi N, Treaba CA, Barletta V, Herranz E, Mehndiratta A et al (2024) A translational MRI approach to validate acute axonal damage detection as an early event in multiple sclerosis. Elife 13:e79169. 10.7554/eLife.79169 38192199 10.7554/eLife.79169 PMC10776086

[31] Chapman TW, Olveda GE, Bame X, Pereira E, Hill RA (2023) Oligodendrocyte death initiates synchronous remyelination to restore cortical myelin patterns in mice. Nat Neurosci 26:555–569. 10.1038/s41593-023-01271-1 36928635 10.1038/s41593-023-01271-1 PMC10208560

[32] Chen G, Park C-K, Xie R-G, Berta T, Nedergaard M, Ji R-R (2014) Connexin-43 induces chemokine release from spinal cord astrocytes to maintain late-phase neuropathic pain in mice. Brain 137:2193–2209. 10.1093/brain/awu140 24919967 10.1093/brain/awu140 PMC4107738

[33] Chen JQA, McNamara NB, Engelenburg HJ, Jongejan A, Wever DD, Hopman K et al (2024) Distinct transcriptional changes distinguish efficient and poor remyelination in multiple sclerosis. Brain 148:2201–2217. 10.1093/brain/awae414 10.1093/brain/awae414 PMC12129743 39718981

[34] Chen Z, Zhong D, Li G (2019) The role of microglia in viral encephalitis: a review. J Neuroinflammation 16:76. 10.1186/s12974-019-1443-2 30967139 10.1186/s12974-019-1443-2 PMC6454758

[35] Clark IC, Wheeler MA, Lee H-G, Li Z, Sanmarco LM, Thaploo S et al (2023) Identification of astrocyte regulators by nucleic acid cytometry. Nature 614:326–333. 10.1038/s41586-022-05613-0 36599367 10.1038/s41586-022-05613-0 PMC9980163

[36] Clark KC, Josephson A, Benusa SD, Hartley RK, Baer M, Thummala S et al (2016) Compromised axon initial segment integrity in EAE is preceded by microglial reactivity and contact. Glia 64:1190–1209. 10.1002/glia.22991 27100937 10.1002/glia.22991

[37] Clarke J-PWE, Thibault PA, Salapa HE, Kim DE, Hutchinson C, Levin MC (2021) Multiple sclerosis-associated hnRNPA1 mutations alter hnRNPA1 dynamics and influence stress granule formation. Int J Mol Sci 22:2909. 10.3390/ijms22062909 33809384 10.3390/ijms22062909 PMC7998649

[38] Clarkson BD, Grund EM, Standiford MM, Mirchia K, Westphal MS, Muschler LS et al (2023) CD8+ T cells recognizing a neuron-restricted antigen injure axons in a model of multiple sclerosis. J Clin Invest 133:e162788. 10.1172/JCI162788 37676734 10.1172/JCI162788 PMC10617772

[39] Clayton BLL, Barbar L, Sapar M, Kalpana K, Rao C, Migliori B et al (2024) Patient iPSC models reveal glia-intrinsic phenotypes in multiple sclerosis. Cell Stem Cell 31:1701-1713.e8. 10.1016/j.stem.2024.08.002 39191254 10.1016/j.stem.2024.08.002 PMC11560525

[40] Cohen CCH, Popovic MA, Klooster J, Weil M-T, Möbius W, Nave K-A et al (2020) Saltatory conduction along myelinated axons involves a periaxonal nanocircuit. Cell 180:311-322.e15. 10.1016/j.cell.2019.11.039 31883793 10.1016/j.cell.2019.11.039 PMC6978798

[41] Cserép C, Pósfai B, Lénárt N, Fekete R, László ZI, Lele Z et al (2020) Microglia monitor and protect neuronal function through specialized somatic purinergic junctions. Science 367:528–537. 10.1126/science.aax6752 31831638 10.1126/science.aax6752

[42] De Keyser J, Steen C, Mostert JP, Koch MW (2008) Hypoperfusion of the cerebral white matter in multiple sclerosis: possible mechanisms and pathophysiological significance. J Cereb Blood Flow Metab 28:1645–1651. 10.1038/jcbfm.2008.72 18594554 10.1038/jcbfm.2008.72

[43] De Keyser J, Wilczak N, Leta R, Streetland C (1999) Astrocytes in multiple sclerosis lack beta-2 adrenergic receptors. Neurology 53(8):1628–1628. 10.1212/WNL.53.8.1628 10563603 10.1212/wnl.53.8.1628

[44] De Santis S, Granberg T, Ouellette R, Treaba CA, Herranz E, Fan Q et al (2019) Evidence of early microstructural white matter abnormalities in multiple sclerosis from multi-shell diffusion MRI. NeuroImage Clin 22:101699. 10.1016/j.nicl.2019.101699 30739842 10.1016/j.nicl.2019.101699 PMC6370560

[45] Dejanovic B, Wu T, Tsai M-C, Graykowski D, Gandham VD, Rose CM et al (2022) Complement C1q-dependent excitatory and inhibitory synapse elimination by astrocytes and microglia in Alzheimer’s disease mouse models. Nat Aging 2:837–850. 10.1038/s43587-022-00281-1 37118504 10.1038/s43587-022-00281-1 PMC10154216

[46] Doyle V, Virji S, Crompton M (1999) Evidence that cyclophilin-A protects cells against oxidative stress. Biochem J 341:127–132 10377253 PMC1220338

[47] Duncan ID, Radcliff AB, Heidari M, Kidd G, August BK, Wierenga LA (2018) The adult oligodendrocyte can participate in remyelination. Proc Natl Acad Sci USA 115:E11807–E11816. 10.1073/pnas.1808064115 30487224 10.1073/pnas.1808064115 PMC6294923

[48] Dutta DJ, Woo DH, Lee PR, Pajevic S, Bukalo O, Huffman WC et al (2018) Regulation of myelin structure and conduction velocity by perinodal astrocytes. Proc Natl Acad Sci USA 115:11832–11837. 10.1073/pnas.1811013115 30373833 10.1073/pnas.1811013115 PMC6243273

[49] Dutta R, Trapp BD (2007) Pathogenesis of axonal and neuronal damage in multiple sclerosis. Neurology 68:S22-31. 10.1212/01.wnl.0000275229.13012.32 17548565 10.1212/01.wnl.0000275229.13012.32

[50] Enz LS, Zeis T, Schmid D, Geier F, Van Der Meer F, Steiner G et al (2020) Increased HLA-DR expression and cortical demyelination in MS links with HLA-DR15. Neurol Neuroimmunol Neuroinflamm 7:e656. 10.1212/NXI.0000000000000656 31882398 10.1212/NXI.0000000000000656 PMC6943368

[51] Factor DC, Barbeau AM, Allan KC, Hu LR, Madhavan M, Hoang AT et al (2020) Cell type-specific intralocus interactions reveal oligodendrocyte mechanisms in MS. Cell 181:382-395.e21. 10.1016/j.cell.2020.03.002 32246942 10.1016/j.cell.2020.03.002 PMC7426147

[52] Filbin MT (2003) Myelin-associated inhibitors of axonal regeneration in the adult mammalian CNS. Nat Rev Neurosci 4:703–713. 10.1038/nrn1195 12951563 10.1038/nrn1195

[53] Floriddia EM, Lourenço T, Zhang S, van Bruggen D, Hilscher MM, Kukanja P et al (2020) Distinct oligodendrocyte populations have spatial preference and different responses to spinal cord injury. Nat Commun 11:5860. 10.1038/s41467-020-19453-x 33203872 10.1038/s41467-020-19453-x PMC7673029

[54] Franceschi C, Bonafè M, Valensin S, Olivieri F, De Luca M, Ottaviani E et al (2000) Inflamm-aging. An evolutionary perspective on immunosenescence. Ann NY Acad Sci 908:244–254. 10.1111/j.1749-6632.2000.tb06651.x 10911963 10.1111/j.1749-6632.2000.tb06651.x

[55] Franceschi C, Garagnani P, Parini P, Giuliani C, Santoro A (2018) Inflammaging: a new immune–metabolic viewpoint for age-related diseases. Nat Rev Endocrinol 14:576–590. 10.1038/s41574-018-0059-4 30046148 10.1038/s41574-018-0059-4

[56] Frank MG, Barrientos RM, Biedenkapp JC, Rudy JW, Watkins LR, Maier SF (2006) mRNA up-regulation of MHC II and pivotal pro-inflammatory genes in normal brain aging. Neurobiol Aging 27:717–722. 10.1016/j.neurobiolaging.2005.03.013 15890435 10.1016/j.neurobiolaging.2005.03.013

[57] Fransen NL, de Jong BA, Heß K, Kuhlmann T, Vincenten MCJ, Hamann J et al (2021) Absence of B cells in brainstem and white matter lesions associates with less severe disease and absence of oligoclonal bands in MS. Neurol Neuroimmunol Neuroinflamm 8:e955. 10.1212/NXI.0000000000000955 33504635 10.1212/NXI.0000000000000955 PMC7862088

[58] Fünfschilling U, Supplie LM, Mahad D, Boretius S, Saab AS, Edgar J et al (2012) Glycolytic oligodendrocytes maintain myelin and long-term axonal integrity. Nature 485:517–521. 10.1038/nature11007 22622581 10.1038/nature11007 PMC3613737

[59] Gallego-Delgado P, James R, Browne E, Meng J, Umashankar S, Tan L et al (2020) Neuroinflammation in the normal-appearing white matter (NAWM) of the multiple sclerosis brain causes abnormalities at the nodes of Ranvier. PLoS Biol 18:e3001008. 10.1371/journal.pbio.3001008 33315860 10.1371/journal.pbio.3001008 PMC7769608

[60] Gay FW (2006) Early cellular events in multiple sclerosis. Intimations of an extrinsic myelinolytic antigen. Clin Neurol Neurosurg 108:234–240. 10.1016/j.clineuro.2005.11.005 16364541 10.1016/j.clineuro.2005.11.005

[61] Ge Y, Grossman RI, Babb JS, He J, Mannon LJ (2003) Dirty-appearing white matter in multiple sclerosis: volumetric MR imaging and magnetization transfer ratio histogram analysis. AJNR Am J Neuroradiol 24:1935–1940 14625213 PMC8148922

[62] Giannetti P, Politis M, Su P, Turkheimer FE, Malik O, Keihaninejad S et al (2015) Increased PK11195-PET binding in normal-appearing white matter in clinically isolated syndrome. Brain 138:110–119. 10.1093/brain/awu331 25416179 10.1093/brain/awu331 PMC4383265

[63] Gilmore CP, DeLuca GC, Bö L, Owens T, Lowe J, Esiri MM et al (2009) Spinal cord neuronal pathology in multiple sclerosis. Brain Pathol 19:642–649. 10.1111/j.1750-3639.2008.00228.x 19170682 10.1111/j.1750-3639.2008.00228.x PMC8094763

[64] Giovannoni G, Popescu V, Wuerfel J, Hellwig K, Iacobaeus E, Jensen MB et al (2022) Smouldering multiple sclerosis: the ‘real MS.’ Ther Adv Neurol Disord 15:175628642110667. 10.1177/17562864211066751 10.1177/17562864211066751 PMC8793117 35096143

[65] Göbel K, Kraft P, Pankratz S, Gross CC, Korsukewitz C, Kwiecien R et al (2016) Prothrombin and factor X are elevated in multiple sclerosis patients. Ann Neurol 80:946–951. 10.1002/ana.24807 27774643 10.1002/ana.24807

[66] Gordon GRJ, Mulligan SJ, MacVicar BA (2007) Astrocyte control of the cerebrovasculature. Glia 55:1214–1221. 10.1002/glia.20543 17659528 10.1002/glia.20543

[67] Guillemin C, Vandeleene N, Charonitis M, Requier F, Delrue G, Lommers E et al (2024) Brain microstructure is linked to cognitive fatigue in early multiple sclerosis. J Neurol 271:3537–3545. 10.1007/s00415-024-12316-1 38538776 10.1007/s00415-024-12316-1

[68] Hamada MS, Kole MHP (2015) Myelin loss and axonal ion channel adaptations associated with gray matter neuronal hyperexcitability. J Neurosci 35:7272–7286. 10.1523/JNEUROSCI.4747-14.2015 25948275 10.1523/JNEUROSCI.4747-14.2015 PMC4420788

[69] Harrison DM, Wang KY, Fiol J, Naunton K, Royal W, Hua J et al (2017) Leptomeningeal enhancement at 7T in multiple sclerosis: frequency, morphology, and relationship to cortical volume. J Neuroimaging 27:461–468. 10.1111/jon.12444 28464368 10.1111/jon.12444 PMC5581238

[70] Hauser SL, Cree BAC (2020) Treatment of multiple sclerosis: a review. Am J Med 133:1380-1390.e2. 10.1016/j.amjmed.2020.05.049 32682869 10.1016/j.amjmed.2020.05.049 PMC7704606

[71] Hayakawa K, Esposito E, Wang X, Terasaki Y, Liu Y, Xing C et al (2016) Transfer of mitochondria from astrocytes to neurons after stroke. Nature 535:551–555. 10.1038/nature18928 27466127 10.1038/nature18928 PMC4968589

[72] Healy LM, Zia S, Plemel JR (2022) Towards a definition of microglia heterogeneity. Commun Biol 5:1114. 10.1038/s42003-022-04081-6 36266565 10.1038/s42003-022-04081-6 PMC9585025

[73] Hendrickx DAE, Schuurman KG, van Draanen M, Hamann J, Huitinga I (2014) Enhanced uptake of multiple sclerosis-derived myelin by THP-1 macrophages and primary human microglia. J Neuroinflammation 11:64. 10.1186/1742-2094-11-64 24684721 10.1186/1742-2094-11-64 PMC4108133

[74] Hill RA, Li AM, Grutzendler J (2018) Lifelong cortical myelin plasticity and age-related degeneration in the live mammalian brain. Nat Neurosci 21:683–695. 10.1038/s41593-018-0120-6 29556031 10.1038/s41593-018-0120-6 PMC5920745

[75] Holmes RD, Vavasour IM, Greenfield J, Zhao G, Lee JS, Moore GRW et al (2021) Nonlesional diffusely abnormal appearing white matter in clinically isolated syndrome: prevalence, association with clinical and MRI features, and risk for conversion to multiple sclerosis. J Neuroimaging 31:981–994. 10.1111/jon.12900 34128576 10.1111/jon.12900

[76] Hortega PR (1928) Tercera aportación al conocimiento morfológico e interpretación funcional de la oligodendroglía. Mem Real Soc Esp Hist Nat 14:5–22

[77] Howell OW, Reeves CA, Nicholas R, Carassiti D, Radotra B, Gentleman SM et al (2011) Meningeal inflammation is widespread and linked to cortical pathology in multiple sclerosis. Brain 134:2755–2771. 10.1093/brain/awr182 21840891 10.1093/brain/awr182

[78] Howell WO, Rundle LJ, Garg A, Komada M, Brophy JP, Reynolds R (2010) Activated microglia mediate axo-glial disruption that contributes to axonal injury in multiple sclerosis. J Neuropathol Exp Neurol 69:1017–1033. 10.1097/NEN.0b013e3181f3a5b1 20838243 10.1097/NEN.0b013e3181f3a5b1 PMC4335193

[79] Huiskamp M, Kiljan S, Kulik S, Witte ME, Jonkman LE, Gjm Bol J et al (2022) Inhibitory synaptic loss drives network changes in multiple sclerosis: an ex vivo to in silico translational study. Mult Scler 28:2010–2019. 10.1177/13524585221125381 36189828 10.1177/13524585221125381 PMC9574900

[80] International Multiple Sclerosis Genetics Consortium, Wellcome Trust Case Control Consortium 2, Sawcer S, Hellenthal G, Pirinen M, Spencer CCA, Patsopoulos NA et al (2011) Genetic risk and a primary role for cell-mediated immune mechanisms in multiple sclerosis. Nature 476:214–219. 10.1038/nature10251 21833088 10.1038/nature10251 PMC3182531

[81] Jäkel S, Agirre E, Mendanha Falcão A, Van Bruggen D, Lee KW, Knuesel I et al (2019) Altered human oligodendrocyte heterogeneity in multiple sclerosis. Nature 566:543–547. 10.1038/s41586-019-0903-2 30747918 10.1038/s41586-019-0903-2 PMC6544546

[82] Jakimovski D, Bittner S, Zivadinov R, Morrow SA, Benedict RH, Zipp F et al (2024) Multiple sclerosis. Lancet 403:183–202. 10.1016/S0140-6736(23)01473-3 37949093 10.1016/S0140-6736(23)01473-3

[83] Joost S, Schweiger F, Pfeiffer F, Ertl C, Keiler J, Frank M et al (2022) Cuprizone intoxication results in myelin vacuole formation. Front Cell Neurosci 16:709596. 10.3389/fncel.2022.709596 35250482 10.3389/fncel.2022.709596 PMC8895267

[84] Jürgens T, Jafari M, Kreutzfeldt M, Bahn E, Brück W, Kerschensteiner M et al (2016) Reconstruction of single cortical projection neurons reveals primary spine loss in multiple sclerosis. Brain 139:39–46. 10.1093/brain/awv353 26667278 10.1093/brain/awv353

[85] Kapell H, Fazio L, Dyckow J, Schwarz S, Cruz-Herranz A, Mayer C et al (2023) Neuron-oligodendrocyte potassium shuttling at nodes of ranvier protects against inflammatory demyelination. J Clin Invest 133:e164223. 10.1172/JCI164223 36719741 10.1172/JCI164223 PMC10065072

[86] KapposButzkuevenWiendlSpelmanPellegriniChen LHHTFY et al (2018) Greater sensitivity to multiple sclerosis disability worsening and progression events using a roving versus a fixed reference value in a prospective cohort study. Mult Scler 24:963–973. 10.1177/1352458517709619 28554238 10.1177/1352458517709619 PMC6029149

[87] Kappos L, Wolinsky JS, Giovannoni G, Arnold DL, Wang Q, Bernasconi C et al (2020) Contribution of relapse-independent progression vs relapse-associated worsening to overall confirmed disability accumulation in typical relapsing multiple sclerosis in a pooled analysis of 2 randomized clinical trials. JAMA Neurol 77:1132–1140. 10.1001/jamaneurol.2020.1568 32511687 10.1001/jamaneurol.2020.1568 PMC7281382

[88] Karttunen MJ, Czopka T, Goedhart M, Early JJ, Lyons DA (2017) Regeneration of myelin sheaths of normal length and thickness in the zebrafish CNS correlates with growth of axons in caliber. PLoS ONE 12:e0178058. 10.1371/journal.pone.0178058 28542521 10.1371/journal.pone.0178058 PMC5444792

[89] Kastriti ME, Sargiannidou I, Kleopa KA, Karagogeos D (2015) Differential modulation of the juxtaparanodal complex in multiple sclerosis. Mol Cell Neurosci 67:93–103. 10.1016/j.mcn.2015.06.005 26070930 10.1016/j.mcn.2015.06.005

[90] Kent SA, Miron VE (2024) Microglia regulation of central nervous system myelin health and regeneration. Nat Rev Immunol 24:49–63. 10.1038/s41577-023-00907-4 37452201 10.1038/s41577-023-00907-4

[91] Keough MB, Rogers JA, Zhang P, Jensen SK, Stephenson EL, Chen T et al (2016) An inhibitor of chondroitin sulfate proteoglycan synthesis promotes central nervous system remyelination. Nat Commun 7:11312. 10.1038/ncomms11312 27115988 10.1038/ncomms11312 PMC4853428

[92] Kerkering J, Muinjonov B, Rosiewicz KS, Diecke S, Biese C, Schiweck J et al (2023) *iPSC*-derived reactive astrocytes from patients with multiple sclerosis protect cocultured neurons in inflammatory conditions. J Clin Invest 133:e164637. 10.1172/JCI164637 37219933 10.1172/JCI164637 PMC10313373

[93] Kessler W, Thomas C, Kuhlmann T (2023) Microglia activation in periplaque white matter in multiple sclerosis depends on age and lesion type, but does not correlate with oligodendroglial loss. Acta Neuropathol 146:817–828. 10.1007/s00401-023-02645-2 37897549 10.1007/s00401-023-02645-2 PMC10628007

[94] Khormi I, Al-iedani O, Alshehri A, Ramadan S, Lechner-Scott J (2023) MR myelin imaging in multiple sclerosis: a scoping review. J Neurol Sci 455:122807. 10.1016/j.jns.2023.122807 38035651 10.1016/j.jns.2023.122807

[95] Kiljan S, Preziosa P, Jonkman LE, Van De Berg WD, Twisk J, Pouwels PJ et al (2021) Cortical axonal loss is associated with both gray matter demyelination and white matter tract pathology in progressive multiple sclerosis: evidence from a combined MRI-histopathology study. Mult Scler 27:380–390. 10.1177/1352458520918978 32390507 10.1177/1352458520918978 PMC7897796

[96] Kim JK, Mastronardi FG, Wood DD, Lubman DM, Zand R, Moscarello MA (2003) Multiple sclerosis. Mol Cell Proteomics 2:453–462. 10.1074/mcp.M200050-MCP200 12832457 10.1074/mcp.M200050-MCP200

[97] Klaver R, Popescu V, Voorn P, Galis-de Graaf Y, Van Der Valk P, De Vries HE et al (2015) Neuronal and axonal loss in normal-appearing gray matter and subpial lesions in multiple sclerosis. J Neuropathol Exp Neurol 74:453–458. 10.1097/NEN.0000000000000189 25853695 10.1097/NEN.0000000000000189

[98] Kole K, Voesenek BJB, Brinia ME, Petersen N, Kole MHP (2022) Parvalbumin basket cell myelination accumulates axonal mitochondria to internodes. Nat Commun 13:7598. 10.1038/s41467-022-35350-x 36494349 10.1038/s41467-022-35350-x PMC9734141

[99] Kole MHP, Stuart GJ (2012) Signal processing in the axon initial segment. Neuron 73:235–247. 10.1016/j.neuron.2012.01.007 22284179 10.1016/j.neuron.2012.01.007

[100] Kooi E-J, Strijbis EMM, Van Der Valk P, Geurts JJG (2012) Heterogeneity of cortical lesions in multiple sclerosis: clinical and pathologic implications. Neurology 79:1369–1376. 10.1212/WNL.0b013e31826c1b1c 22972651 10.1212/WNL.0b013e31826c1b1c

[101] Kotter MR, Li W-W, Zhao C, Franklin RJM (2006) Myelin impairs CNS remyelination by inhibiting oligodendrocyte precursor cell differentiation. J Neurosci 26:328–332. 10.1523/JNEUROSCI.2615-05.2006 16399703 10.1523/JNEUROSCI.2615-05.2006 PMC6674302

[102] Kuhlmann T, Moccia M, Coetzee T, Cohen JA, Correale J, Graves J et al (2023) Multiple sclerosis progression: time for a new mechanism-driven framework. Lancet Neurol 22:78–88. 10.1016/S1474-4422(22)00289-7 36410373 10.1016/S1474-4422(22)00289-7 PMC10463558

[103] Kular L, Klose D, Urdánoz-Casado A, Ewing E, Planell N, Gomez-Cabrero D et al (2022) Epigenetic clock indicates accelerated aging in glial cells of progressive multiple sclerosis patients. Front Aging Neurosci 14:926468. 10.3389/fnagi.2022.926468 36092807 10.3389/fnagi.2022.926468 PMC9454196

[104] Kutzelnigg A, Lucchinetti CF, Stadelmann C, Brück W, Rauschka H, Bergmann M et al (2005) Cortical demyelination and diffuse white matter injury in multiple sclerosis. Brain 128:2705–2712. 10.1093/brain/awh641 16230320 10.1093/brain/awh641

[105] Lassmann H (2018) Multiple sclerosis pathology. Cold Spring Harb Perspect Med 8:a028936. 10.1101/cshperspect.a028936 29358320 10.1101/cshperspect.a028936 PMC5830904

[106] Lee M-H, Perl DP, Nair G, Li W, Maric D, Murray H et al (2021) Microvascular injury in the brains of patients with Covid-19. N Engl J Med 384:481–483. 10.1056/NEJMc2033369 33378608 10.1056/NEJMc2033369 PMC7787217

[107] Lie IA, Weeda MM, Mattiesing RM, Mol MAE, Pouwels PJW, Barkhof F et al (2022) Relationship between white matter lesions and gray matter atrophy in multiple sclerosis: a systematic review. Neurology 98:e1532–e1545. 10.1212/WNL.0000000000200006 10.1212/WNL.0000000000200006 PMC9038199 35173016

[108] Liu Y, Given KS, Harlow DE, Matschulat AM, Macklin WB, Bennett JL et al (2017) Myelin-specific multiple sclerosis antibodies cause complement-dependent oligodendrocyte loss and demyelination. Acta Neuropathol Commun 5:25. 10.1186/s40478-017-0428-6 28340598 10.1186/s40478-017-0428-6 PMC5366134

[109] Liu YU, Ying Y, Li Y, Eyo UB, Chen T, Zheng J et al (2019) Neuronal network activity controls microglial process surveillance in awake mice via norepinephrine signaling. Nat Neurosci 22:1771–1781. 10.1038/s41593-019-0511-3 31636449 10.1038/s41593-019-0511-3 PMC6858573

[110] Lohrberg M, Winkler A, Franz J, van der Meer F, Ruhwedel T, Sirmpilatze N et al (2020) Lack of astrocytes hinders parenchymal oligodendrocyte precursor cells from reaching a myelinating state in osmolyte-induced demyelination. Acta Neuropathol Commun 8:224. 10.1186/s40478-020-01105-2 33357244 10.1186/s40478-020-01105-2 PMC7761156

[111] Lublin FD, Häring DA, Ganjgahi H, Ocampo A, Hatami F, Čuklina J et al (2022) How patients with multiple sclerosis acquire disability. Brain 145:3147–3161. 10.1093/brain/awac016 35104840 10.1093/brain/awac016 PMC9536294

[112] Lucchinetti C, Brück W, Parisi J, Scheithauer B, Rodriguez M, Lassmann H (2000) Heterogeneity of multiple sclerosis lesions: implications for the pathogenesis of demyelination. Ann Neurol 47:707–717. 10.1002/1531-8249(200006)47:6%3c707::aid-ana3%3e3.0.co;2-q 10852536 10.1002/1531-8249(200006)47:6<707::aid-ana3>3.0.co;2-q

[113] Luchicchi A, Hart B, Frigerio I, Van Dam A, Perna L, Offerhaus HL et al (2021) Axon-myelin unit blistering as early event in normal appearing white matter. Ann Neurol 89:711–725. 10.1002/ana.26014 33410190 10.1002/ana.26014 PMC8048993

[114] Luchicchi A, Muñoz-Gonzalez G, Halperin ST, Strijbis E, Van Dijk LHM, Foutiadou C et al (2024) Micro-diffusely abnormal white matter: an early multiple sclerosis lesion phase with intensified myelin blistering. Ann Clin Transl Neurol 11:973–988. 10.1002/acn3.52015 38425098 10.1002/acn3.52015 PMC11021636

[115] Machado-Santos J, Saji E, Tröscher AR, Paunovic M, Liblau R, Gabriely G et al (2018) The compartmentalized inflammatory response in the multiple sclerosis brain is composed of tissue-resident CD8+ T lymphocytes and B cells. Brain 141:2066–2082. 10.1093/brain/awy151 29873694 10.1093/brain/awy151 PMC6022681

[116] Macnair W, Calini D, Agirre E, Bryois J, Jäkel S, Smith RS et al (2024) snRNA-seq stratifies multiple sclerosis patients into distinct white matter glial responses. Neuron. 10.1016/j.neuron.2024.11.016 39708806 10.1016/j.neuron.2024.11.016

[117] Maglione M, Tress O, Haas B, Karram K, Trotter J, Willecke K et al (2010) Oligodendrocytes in mouse corpus callosum are coupled via gap junction channels formed by connexin47 and connexin32. Glia 58:1104–1117. 10.1002/glia.20991 20468052 10.1002/glia.20991

[118] Magliozzi R, Howell OW, Calabrese M, Reynolds R (2023) Meningeal inflammation as a driver of cortical grey matter pathology and clinical progression in multiple sclerosis. Nat Rev Neurol 19:461–476. 10.1038/s41582-023-00838-7 37400550 10.1038/s41582-023-00838-7

[119] Magliozzi R, Howell OW, Reeves C, Roncaroli F, Nicholas R, Serafini B et al (2010) A gradient of neuronal loss and meningeal inflammation in multiple sclerosis. Ann Neurol 68:477–493. 10.1002/ana.22230 20976767 10.1002/ana.22230

[120] Marisca R, Hoche T, Agirre E, Hoodless LJ, Barkey W, Auer F et al (2020) Functionally distinct subgroups of oligodendrocyte precursor cells integrate neural activity and execute myelin formation. Nat Neurosci 23:363–374. 10.1038/s41593-019-0581-2 32066987 10.1038/s41593-019-0581-2 PMC7292734

[121] Markoullis K, Sargiannidou I, Schiza N, Roncaroli F, Reynolds R, Kleopa KA (2014) Oligodendrocyte gap junction loss and disconnection from reactive astrocytes in multiple sclerosis gray matter. J Neuropathol Exp Neurol 73:865–879. 10.1097/NEN.0000000000000106 25101702 10.1097/NEN.0000000000000106

[122] Marques S, Zeisel A, Codeluppi S, van Bruggen D, Mendanha Falcão A, Xiao L et al (2016) Oligodendrocyte heterogeneity in the mouse juvenile and adult central nervous system. Science 352:1326–1329. 10.1126/science.aaf6463 27284195 10.1126/science.aaf6463 PMC5221728

[123] Marschallinger J, Iram T, Zardeneta M, Lee SE, Lehallier B, Haney MS et al (2020) Lipid-droplet-accumulating microglia represent a dysfunctional and proinflammatory state in the aging brain. Nat Neurosci 23:194–208. 10.1038/s41593-019-0566-1 31959936 10.1038/s41593-019-0566-1 PMC7595134

[124] Mastronardi FG, Noor A, Wood DD, Paton T, Moscarello MA (2007) Peptidyl argininedeiminase 2 CpG island in multiple sclerosis white matter is hypomethylated. J Neurosci Res 85:2006–2016. 10.1002/jnr.21329 17469138 10.1002/jnr.21329

[125] Mathey EK, Derfuss T, Storch MK, Williams KR, Hales K, Woolley DR et al (2007) Neurofascin as a novel target for autoantibody-mediated axonal injury. J Exp Med 204:2363–2372. 10.1084/jem.20071053 17846150 10.1084/jem.20071053 PMC2118456

[126] McKenzie IA, Ohayon D, Li H, de Faria JP, Emery B, Tohyama K et al (2014) Motor skill learning requires active central myelination. Science 346:318–322. 10.1126/science.1254960 25324381 10.1126/science.1254960 PMC6324726

[127] McKeon RJ, Schreiber RC, Rudge JS, Silver J (1991) Reduction of neurite outgrowth in a model of glial scarring following CNS injury is correlated with the expression of inhibitory molecules on reactive astrocytes. J Neurosci 11:3398–3411. 10.1523/JNEUROSCI.11-11-03398.1991 1719160 10.1523/JNEUROSCI.11-11-03398.1991 PMC6575543

[128] McNamara NB, Munro DAD, Bestard-Cuche N, Uyeda A, Bogie JFJ, Hoffmann A et al (2023) Microglia regulate central nervous system myelin growth and integrity. Nature 613:120–129. 10.1038/s41586-022-05534-y 36517604 10.1038/s41586-022-05534-y PMC9812791

[129] Meschkat M, Steyer AM, Weil M-T, Kusch K, Jahn O, Piepkorn L et al (2022) White matter integrity in mice requires continuous myelin synthesis at the inner tongue. Nat Commun 13:1163. 10.1038/s41467-022-28720-y 35246535 10.1038/s41467-022-28720-y PMC8897471

[130] Messmer ML, Salapa HE, Popescu BF, Levin MC (2024) RNA-binding protein dysfunction links smoldering/slowly expanding lesions to neurodegeneration in multiple sclerosis. Ann Neurol. 10.1002/ana.27114 39422285 10.1002/ana.27114

[131] Meyer N, Richter N, Fan Z, Siemonsmeier G, Pivneva T, Jordan P et al (2018) Oligodendrocytes in the mouse corpus callosum maintain axonal function by delivery of glucose. Cell Rep 22:2383–2394. 10.1016/j.celrep.2018.02.022 29490274 10.1016/j.celrep.2018.02.022

[132] Michailidou I, Naessens DMP, Hametner S, Guldenaar W, Kooi E-J, Geurts JJG et al (2017) Complement C3 on microglial clusters in multiple sclerosis occur in chronic but not acute disease: implication for disease pathogenesis: complement C3 and microglial clusters in MS. Glia 65:264–277. 10.1002/glia.23090 27778395 10.1002/glia.23090 PMC5215693

[133] Micheva KD, Wolman D, Mensh BD, Pax E, Buchanan J, Smith SJ et al (2016) A large fraction of neocortical myelin ensheathes axons of local inhibitory neurons. Elife 5:e15784. 10.7554/eLife.15784 27383052 10.7554/eLife.15784 PMC4972537

[134] Mishra MK, Wang J, Keough MB, Fan Y, Silva C, Sloka S et al (2014) Laquinimod reduces neuroaxonal injury through inhibiting microglial activation. Ann Clin Transl Neurol 1:409–422. 10.1002/acn3.67 25356411 10.1002/acn3.67 PMC4184669

[135] Moll NM, Rietsch AM, Thomas S, Ransohoff AJ, Lee J-C, Fox R et al (2011) Multiple sclerosis normal-appearing white matter: pathology-imaging correlations. Ann Neurol 70:764–773. 10.1002/ana.22521 22162059 10.1002/ana.22521 PMC3241216

[136] Moore CS, Cui Q-L, Warsi NM, Durafourt BA, Zorko N, Owen DR et al (2015) Direct and indirect effects of immune and central nervous system-resident cells on human oligodendrocyte progenitor cell differentiation. J Immunol 194:761–772. 10.4049/jimmunol.1401156 25505283 10.4049/jimmunol.1401156

[137] Moscarello MA, Lei H, Mastronardi FG, Winer S, Tsui H, Li Z et al (2013) Inhibition of peptidyl-arginine deiminases reverses protein-hypercitrullination and disease in mouse models of multiple sclerosis. Dis Model Mech 6:467–478. 10.1242/dmm.010520 23118341 10.1242/dmm.010520 PMC3597028

[138] Nave K-A (2010) Myelination and the trophic support of long axons. Nat Rev Neurosci 11:275–283. 10.1038/nrn2797 20216548 10.1038/nrn2797

[139] Nave K-A, Asadollahi E, Sasmita A (2023) Expanding the function of oligodendrocytes to brain energy metabolism. Curr Opin Neurobiol 83:102782. 10.1016/j.conb.2023.102782 37703600 10.1016/j.conb.2023.102782

[140] Nebeling FC, Poll S, Justus LC, Steffen J, Keppler K, Mittag M et al (2023) Microglial motility is modulated by neuronal activity and correlates with dendritic spine plasticity in the hippocampus of awake mice. Elife 12:e83176. 10.7554/eLife.83176 36749020 10.7554/eLife.83176 PMC9946443

[141] Neely SA, Williamson JM, Klingseisen A, Zoupi L, Early JJ, Williams A et al (2022) New oligodendrocytes exhibit more abundant and accurate myelin regeneration than those that survive demyelination. Nat Neurosci 25:415–420. 10.1038/s41593-021-01009-x 35165460 10.1038/s41593-021-01009-x PMC7612594

[142] van Nierop GP, van Luijn MM, Michels SS, Melief M-J, Janssen M, Langerak AW et al (2017) Phenotypic and functional characterization of T cells in white matter lesions of multiple sclerosis patients. Acta Neuropathol 134:383–401. 10.1007/s00401-017-1744-4 28624961 10.1007/s00401-017-1744-4 PMC5563341

[143] Nimmerjahn A, Kirchhoff F, Helmchen F (2005) Resting microglial cells are highly dynamic surveillants of brain parenchyma in vivo. Science 308:1314–1318. 10.1126/science.1110647 15831717 10.1126/science.1110647

[144] Nowacki P, Koziarska D, Masztalewicz M (2019) Microglia and astroglia proliferation within the normal appearing white matter in histologically active and inactive multiple sclerosis. Folia Neuropathol 57:249–257. 10.5114/fn.2019.88453 31588711 10.5114/fn.2019.88453

[145] Nutma E, Stephenson JA, Gorter RP, De Bruin J, Boucherie DM, Donat CK et al (2019) A quantitative neuropathological assessment of translocator protein expression in multiple sclerosis. Brain 142:3440–3455. 10.1093/brain/awz287 31578541 10.1093/brain/awz287 PMC6821167

[146] Oksenberg JR, Baranzini SE, Sawcer S, Hauser SL (2008) The genetics of multiple sclerosis: SNPs to pathways to pathogenesis. Nat Rev Genet 9:516–526. 10.1038/nrg2395 18542080 10.1038/nrg2395

[147] Oost W, Huitema AJ, Kats K, Giepmans BNG, Kooistra SM, Eggen BJL et al (2023) Pathological ultrastructural alterations of myelinated axons in normal appearing white matter in progressive multiple sclerosis. Acta Neuropathol Commun 11:100. 10.1186/s40478-023-01598-7 37340488 10.1186/s40478-023-01598-7 PMC10283269

[148] Orellana JA, Froger N, Ezan P, Jiang JX, Bennett MVL, Naus CC et al (2011) ATP and glutamate released via astroglial connexin 43 hemichannels mediate neuronal death through activation of pannexin 1 hemichannels. J Neurochem 118:826–840. 10.1111/j.1471-4159.2011.07210.x 21294731 10.1111/j.1471-4159.2011.07210.x PMC3108012

[149] Ousman SS, Tomooka BH, Van Noort JM, Wawrousek EF, O’Conner K, Hafler DA et al (2007) Protective and therapeutic role for αB-crystallin in autoimmune demyelination. Nature 448:474–479. 10.1038/nature05935 17568699 10.1038/nature05935

[150] Owens GP, Fellin TJ, Matschulat A, Salas V, Schaller KL, Given KS et al (2023) Pathogenic myelin-specific antibodies in multiple sclerosis target conformational proteolipid protein 1-anchored membrane domains. J Clin Invest 133:e162731. 10.1172/JCI162731 37561592 10.1172/JCI162731 PMC10541191

[151] Pagani E, Rocca MA, Gallo A, Rovaris M, Martinelli V, Comi G et al (2005) Regional brain atrophy evolves differently in patients with multiple sclerosis according to clinical phenotype. AJNR Am J Neuroradiol 26:341–346 15709132 PMC7974082

[152] Pan S, Mayoral SR, Choi HS, Chan JR, Kheirbek MA (2020) Preservation of a remote fear memory requires new myelin formation. Nat Neurosci 23:487–499. 10.1038/s41593-019-0582-1 32042175 10.1038/s41593-019-0582-1 PMC7213814

[153] Park SJ, Lee H, Jo DS, Jo YK, Shin JH, Kim HB et al (2015) Heterogeneous nuclear ribonucleoprotein A1 post-transcriptionally regulates Drp1 expression in neuroblastoma cells. Biochim Biophys Acta 1849:1423–1431. 10.1016/j.bbagrm.2015.10.017 26518267 10.1016/j.bbagrm.2015.10.017 PMC4655839

[154] Parsons ME, O’Connell K, Allen S, Egan K, Szklanna PB, McGuigan C et al (2017) Thrombin generation correlates with disease duration in multiple sclerosis (MS): novel insights into the MS-associated prothrombotic state. Mult Scler J 3:2055217317747624. 10.1177/2055217317747624 10.1177/2055217317747624 PMC5753921 29318029

[155] Pernin F, Cui Q-L, Mohammadnia A, Fernandes MGF, Hall JA, Srour M et al (2024) Regulation of stress granule formation in human oligodendrocytes. Nat Commun 15(1):1524. 10.1038/s41467-024-45746-6 38374028 10.1038/s41467-024-45746-6 PMC10876533

[156] Peters A (2002) The effects of normal aging on myelin and nerve fibers: a review. J Neurocytol 31:581–593. 10.1023/a:1025731309829 14501200 10.1023/a:1025731309829

[157] Peterson JW, Bö L, Mörk S, Chang A, Trapp BD (2001) Transected neurites, apoptotic neurons, and reduced inflammation in cortical multiple sclerosis lesions. Ann Neurol 50:389–400. 10.1002/ana.1123 11558796 10.1002/ana.1123

[158] Petrova N, Nutma E, Carassiti D, Rs Newman J, Amor S, Altmann DR et al (2020) Synaptic loss in multiple sclerosis spinal cord. Ann Neurol 88:619–625. 10.1002/ana.25835 32608018 10.1002/ana.25835

[159] Pichler A, Khalil M, Langkammer C, Pinter D, Bachmaier G, Ropele S et al (2016) Combined analysis of global and compartmental brain volume changes in early multiple sclerosis in clinical practice. Mult Scler 22:340–346. 10.1177/1352458515593405 26163072 10.1177/1352458515593405

[160] Poon KWC, Brideau C, Klaver R, Schenk GJ, Geurts JJ, Stys PK (2018) Lipid biochemical changes detected in normal appearing white matter of chronic multiple sclerosis by spectral coherent Raman imaging. Chem Sci 9:1586–1595. 10.1039/C7SC03992A 29675203 10.1039/c7sc03992a PMC5890326

[161] Portaccio E, Bellinvia A, Fonderico M, Pastò L, Razzolini L, Totaro R et al (2022) Progression is independent of relapse activity in early multiple sclerosis: a real-life cohort study. Brain 145:2796–2805. 10.1093/brain/awac111 35325059 10.1093/brain/awac111

[162] Rahmanzadeh R, Lu P-J, Barakovic M, Weigel M, Maggi P, Nguyen TD et al (2021) Myelin and axon pathology in multiple sclerosis assessed by myelin water and multi-shell diffusion imaging. Brain 144:1684–1696. 10.1093/brain/awab088 33693571 10.1093/brain/awab088 PMC8374972

[163] Ramaglia V (2021) Complement-associated loss of CA2 inhibitory synapses in the demyelinated hippocampus impairs memory. Acta Neuropathol 142:643–667. 10.1007/s00401-021-02338-8 34170374 10.1007/s00401-021-02338-8 PMC8423657

[164] Ramos-Cabrer P, Cabrera-Zubizarreta A, Padro D, Matute-González M, Rodríguez-Antigüedad A, Matute C (2025) Reversible reduction in brain myelin content upon marathon running. Nat Metab. 10.1038/s42255-025-01244-7 40128612 10.1038/s42255-025-01244-7 PMC12021653

[165] Ritchie JM (1982) On the relation between fibre diameter and conduction velocity in myelinated nerve fibres. Proc R Soc Lond B Biol Sci 217:29–35. 10.1098/rspb.1982.0092 6131421 10.1098/rspb.1982.0092

[166] Romanelli E, Merkler D, Mezydlo A, Weil M-T, Weber MS, Nikić I et al (2016) Myelinosome formation represents an early stage of oligodendrocyte damage in multiple sclerosis and its animal model. Nat Commun 7:13275. 10.1038/ncomms13275 27848954 10.1038/ncomms13275 PMC5116090

[167] Ronzano R, Roux T, Thetiot M, Aigrot MS, Richard L, Lejeune FX et al (2021) Microglia-neuron interaction at nodes of Ranvier depends on neuronal activity through potassium release and contributes to remyelination. Nat Commun 12:5219. 10.1038/s41467-021-25486-7 34471138 10.1038/s41467-021-25486-7 PMC8410814

[168] Rossi D (2015) Astrocyte physiopathology: at the crossroads of intercellular networking, inflammation and cell death. Prog Neurobiol 130:86–120. 10.1016/j.pneurobio.2015.04.003 25930681 10.1016/j.pneurobio.2015.04.003

[169] Roufagalas I, Avloniti M, Fortosi A, Xingi E, Thomaidou D, Probert L et al (2021) Novel cell-based analysis reveals region-dependent changes in microglial dynamics in grey matter in a cuprizone model of demyelination. Neurobiol Dis 157:105449. 10.1016/j.nbd.2021.105449 34274460 10.1016/j.nbd.2021.105449

[170] Saab AS, Tzvetavona ID, Trevisiol A, Baltan S, Dibaj P, Kusch K et al (2016) Oligodendroglial NMDA receptors regulate glucose import and axonal energy metabolism. Neuron 91:119–132. 10.1016/j.neuron.2016.05.016 27292539 10.1016/j.neuron.2016.05.016 PMC9084537

[171] Safaiyan S, Besson-Girard S, Kaya T, Cantuti-Castelvetri L, Liu L, Ji H et al (2021) White matter aging drives microglial diversity. Neuron 109:1100-1117.e10. 10.1016/j.neuron.2021.01.027 33606969 10.1016/j.neuron.2021.01.027

[172] Safaiyan S, Kannaiyan N, Snaidero N, Brioschi S, Biber K, Yona S et al (2016) Age-related myelin degradation burdens the clearance function of microglia during aging. Nat Neurosci 19:995–998. 10.1038/nn.4325 27294511 10.1038/nn.4325 PMC7116794

[173] Sanders V, Conrad AJ, Tourtellotte WW (1993) On classification of post-mortem multiple sclerosis plaques for neuroscientists. J Neuroimmunol 46:207–216. 10.1016/0165-5728(93)90251-s 8360330 10.1016/0165-5728(93)90251-s

[174] Scalfari A, Traboulsee A, Oh J, Airas L, Bittner S, Calabrese M et al (2024) Smouldering-associated worsening in multiple sclerosis: an international consensus statement on definition, biology, clinical implications, and future directions. Ann Neurol 96:826–845. 10.1002/ana.27034 39051525 10.1002/ana.27034

[175] Schäffner E, Bosch-Queralt M, Edgar JM, Lehning M, Strauß J, Fleischer N et al (2023) Myelin insulation as a risk factor for axonal degeneration in autoimmune demyelinating disease. Nat Neurosci 26:1218–1228. 10.1038/s41593-023-01366-9 37386131 10.1038/s41593-023-01366-9 PMC10322724

[176] Schirmer L, Velmeshev D, Holmqvist S, Kaufmann M, Werneburg S, Jung D et al (2019) Neuronal vulnerability and multilineage diversity in multiple sclerosis. Nature 573:75–82. 10.1038/s41586-019-1404-z 31316211 10.1038/s41586-019-1404-z PMC6731122

[177] Schmidt H, Hahn G, Deco G, Knösche TR (2021) Ephaptic coupling in white matter fibre bundles modulates axonal transmission delays. PLoS Comput Biol 17:e1007858. 10.1371/journal.pcbi.1007858 33556058 10.1371/journal.pcbi.1007858 PMC7895385

[178] Seewann A, Vrenken H, Van Der Valk P, Blezer ELA, Knol DL, Castelijns JA et al (2009) Diffusely abnormal white matter in chronic multiple sclerosis: imaging and histopathologic analysis. Arch Neurol. 10.1001/archneurol.2009.57 19433660 10.1001/archneurol.2009.57

[179] Seidl AH, Rubel EW, Harris DM (2010) Mechanisms for adjusting interaural time differences to achieve binaural coincidence detection. J Neurosci 30:70–80. 10.1523/JNEUROSCI.3464-09.2010 20053889 10.1523/JNEUROSCI.3464-09.2010 PMC2822993

[180] Senol AD, Pinto G, Beau M, Guillemot V, Dupree JL, Stadelmann C et al (2022) Alterations of the axon initial segment in multiple sclerosis grey matter. Brain Commun 4:fcac284. 10.1093/braincomms/fcac284 36451656 10.1093/braincomms/fcac284 PMC9700164

[181] Shaharabani R, Ram-On M, Avinery R, Aharoni R, Arnon R, Talmon Y et al (2016) Structural transition in myelin membrane as initiator of multiple sclerosis. J Am Chem Soc 138:12159–12165. 10.1021/jacs.6b04826 27548321 10.1021/jacs.6b04826

[182] Singh S, Metz I, Amor S, Van Der Valk P, Stadelmann C, Brück W (2013) Microglial nodules in early multiple sclerosis white matter are associated with degenerating axons. Acta Neuropathol 125:595–608. 10.1007/s00401-013-1082-0 23354834 10.1007/s00401-013-1082-0 PMC3611040

[183] Singhal T, Cicero S, Pan H, Carter K, Dubey S, Chu R et al (2020) Regional microglial activation in the substantia nigra is linked with fatigue in MS. Neurol Neuroimmunol Neuroinflamm 7:e854. 10.1212/NXI.0000000000000854 32769103 10.1212/NXI.0000000000000854 PMC7643614

[184] Sobel RA, Ahmed AS (2001) White matter extracellular matrix chondroitin sulfate/dermatan sulfate proteoglycans in multiple sclerosis. J Neuropathol Exp Neurol 60:1198–1207. 10.1093/jnen/60.12.1198 11764092 10.1093/jnen/60.12.1198

[185] Steadman PE, Xia F, Ahmed M, Mocle AJ, Penning ARA, Geraghty AC et al (2020) Disruption of oligodendrogenesis impairs memory consolidation in adult mice. Neuron 105:150-164.e6. 10.1016/j.neuron.2019.10.013 31753579 10.1016/j.neuron.2019.10.013 PMC7579726

[186] Stedehouder J, Couey JJ, Brizee D, Hosseini B, Slotman JA, Dirven CMF et al (2017) Fast-spiking parvalbumin interneurons are frequently myelinated in the cerebral cortex of mice and humans. Cereb Cortex 27:5001–5013. 10.1093/cercor/bhx203 28922832 10.1093/cercor/bhx203

[187] Stephenson EL, Jain RW, Ghorbani S, Gorter RP, D’Mello C, Yong VW (2024) Uncovering novel extracellular matrix transcriptome alterations in lesions of multiple sclerosis. IJMS 25:1240. 10.3390/ijms25021240 38279239 10.3390/ijms25021240 PMC10816920

[188] Stys PK (2011) The axo-myelinic synapse. Trends Neurosci 34:393–400. 10.1016/j.tins.2011.06.004 21741098 10.1016/j.tins.2011.06.004

[189] Sucksdorff M, Matilainen M, Tuisku J, Polvinen E, Vuorimaa A, Rokka J et al (2020) Brain TSPO-PET predicts later disease progression independent of relapses in multiple sclerosis. Brain 143:3318–3330. 10.1093/brain/awaa275 33006604 10.1093/brain/awaa275 PMC7719021

[190] Takase EO, Yamasaki R, Nagata S, Watanabe M, Masaki K, Yamaguchi H et al (2024) Astroglial connexin 43 is a novel therapeutic target for chronic multiple sclerosis model. Sci Rep 14:10877. 10.1038/s41598-024-61508-2 38740862 10.1038/s41598-024-61508-2 PMC11091090

[191] Tallantyre EC, Bø L, Al-Rawashdeh O, Owens T, Polman CH, Lowe JS et al (2010) Clinico-pathological evidence that axonal loss underlies disability in progressive multiple sclerosis. Mult Scler 16:406–411. 10.1177/1352458510364992 20215480 10.1177/1352458510364992

[192] Tay TL, Mai D, Dautzenberg J, Fernándezklett F, Lin G, Null S et al (2017) A new fate mapping system reveals context-dependent random or clonal expansion of microglia. Nat Neurosci 20:793–803. 10.1038/nn.4547 28414331 10.1038/nn.4547

[193] Teo W, Caprariello AV, Morgan ML, Luchicchi A, Schenk GJ, Joseph JT et al (2021) Nile red fluorescence spectroscopy reports early physicochemical changes in myelin with high sensitivity. Proc Natl Acad Sci U S A 118:e2016897118. 10.1073/pnas.2016897118 33593907 10.1073/pnas.2016897118 PMC7923366

[194] Thevaranjan N, Puchta A, Schulz C, Naidoo A, Szamosi JC, Verschoor CP et al (2017) Age-associated microbial dysbiosis promotes intestinal permeability, systemic inflammation, and macrophage dysfunction. Cell Host Microbe 21:455-466.e4. 10.1016/j.chom.2017.03.002 28407483 10.1016/j.chom.2017.03.002 PMC5392495

[195] Timmler S, Simons M (2019) Grey matter myelination. Glia 67:2063–2070. 10.1002/glia.23614 30860619 10.1002/glia.23614

[196] Tobin WO, Kalinowska-Lyszczarz A, Weigand SD, Guo Y, Tosakulwong N, Parisi JE et al (2021) Clinical correlation of multiple sclerosis immunopathologic subtypes. Neurology 97:e1906–e1913. 10.1212/WNL.0000000000012782 34504026 10.1212/WNL.0000000000012782 PMC8601208

[197] Tomassy GS, Berger DR, Chen H-H, Kasthuri N, Hayworth KJ, Vercelli A et al (2014) Distinct profiles of myelin distribution along single axons of pyramidal neurons in the neocortex. Science 344:319–324. 10.1126/science.1249766 24744380 10.1126/science.1249766 PMC4122120

[198] Trapp BD, Peterson J, Ransohoff RM, Rudick R, Mörk S, Bö L (1998) Axonal transection in the lesions of multiple sclerosis. N Engl J Med 338:278–285. 10.1056/NEJM199801293380502 9445407 10.1056/NEJM199801293380502

[199] Tremblay M-È, Lowery RL, Majewska AK (2010) Microglial interactions with synapses are modulated by visual experience. PLoS Biol 8:e1000527. 10.1371/journal.pbio.1000527 21072242 10.1371/journal.pbio.1000527 PMC2970556

[200] Tress O, Maglione M, May D, Pivneva T, Richter N, Seyfarth J et al (2012) Panglial gap junctional communication is essential for maintenance of myelin in the CNS. J Neurosci 32:7499–7518. 10.1523/JNEUROSCI.0392-12.2012 22649229 10.1523/JNEUROSCI.0392-12.2012 PMC6703577

[201] Tur C, Carbonell-Mirabent P, Cobo-Calvo Á, Otero-Romero S, Arrambide G, Midaglia L et al (2023) Association of early progression independent of relapse activity with long-term disability after a first demyelinating event in multiple sclerosis. JAMA Neurol 80:151. 10.1001/jamaneurol.2022.4655 36534392 10.1001/jamaneurol.2022.4655 PMC9856884

[202] Tutuncu M, Tang J, Zeid NA, Kale N, Crusan DJ, Atkinson EJ et al (2013) Onset of progressive phase is an age-dependent clinical milestone in multiple sclerosis. Mult Scler 19:188–198. 10.1177/1352458512451510 22736750 10.1177/1352458512451510 PMC4029334

[203] van der Valk P, Amor S (2009) Preactive lesions in multiple sclerosis. Curr Opin Neurol 22:207–213. 10.1097/WCO.0b013e32832b4c76 19417567 10.1097/WCO.0b013e32832b4c76

[204] van der Valk P, De Groot CJ (2000) Staging of multiple sclerosis (MS) lesions: pathology of the time frame of MS. Neuropathol Appl Neurobiol 26:2–10. 10.1046/j.1365-2990.2000.00217.x 10736062 10.1046/j.1365-2990.2000.00217.x

[205] Van Den Bosch AMR, Hümmert S, Steyer A, Ruhwedel T, Hamann J, Smolders J et al (2023) Ultrastructural axon–myelin unit alterations in multiple sclerosis correlate with inflammation. Ann Neurol 93:856–870. 10.1002/ana.26585 36565265 10.1002/ana.26585

[206] Van Den Bosch AMR, Van Der Poel M, Fransen NL, Vincenten MCJ, Bobeldijk AM, Jongejan A et al (2024) Profiling of microglia nodules in multiple sclerosis reveals propensity for lesion formation. Nat Commun 15:1667. 10.1038/s41467-024-46068-3 38396116 10.1038/s41467-024-46068-3 PMC10891081

[207] Van Den Bosch AMR, Wever D, Schonewille P, Schuller SL, Smolders J, Hamann J et al (2024) Cortical CD200–CD200R and CD47–SIRPα expression is associated with multiple sclerosis pathology. Brain Commun 6:fcae264. 10.1093/braincomms/fcae264 39175944 10.1093/braincomms/fcae264 PMC11339711

[208] Van Der Poel M, Ulas T, Mizee MR, Hsiao C-C, Miedema SSM, Adelia SKG et al (2019) Transcriptional profiling of human microglia reveals grey–white matter heterogeneity and multiple sclerosis-associated changes. Nat Commun 10:1139. 10.1038/s41467-019-08976-7 30867424 10.1038/s41467-019-08976-7 PMC6416318

[209] Van Horssen J, Singh S, Van Der Pol S, Kipp M, Lim JL, Peferoen L et al (2012) Clusters of activated microglia in normal-appearing white matter show signs of innate immune activation. J Neuroinflammation 9:156. 10.1186/1742-2094-9-156 22747960 10.1186/1742-2094-9-156 PMC3411485

[210] Van Noort JM, Bsibsi M, Gerritsen WH, Van Der Valk P, Bajramovic JJ, Steinman L et al (2010) Αb-crystallin is a target for adaptive immune responses and a trigger of innate responses in preactive multiple sclerosis lesions. J Neuropathol Exp Neurol 69:694–703. 10.1097/NEN.0b013e3181e4939c 20535035 10.1097/NEN.0b013e3181e4939c

[211] Van Olst L, Rodriguez-Mogeda C, Picon C, Kiljan S, James RE, Kamermans A et al (2021) Meningeal inflammation in multiple sclerosis induces phenotypic changes in cortical microglia that differentially associate with neurodegeneration. Acta Neuropathol 141:881–899. 10.1007/s00401-021-02293-4 33779783 10.1007/s00401-021-02293-4 PMC8113309

[212] Vavasour IM, Becquart P, Gill J, Zhao G, Yik JT, Traboulsee A et al (2022) Diffusely abnormal white matter in clinically isolated syndrome is associated with parenchymal loss and elevated neurofilament levels. Mult Scler Relat Disord 57:103422. 10.1016/j.msard.2021.103422 34871858 10.1016/j.msard.2021.103422

[213] Vercellino M, Marasciulo S, Grifoni S, Vallino-Costassa E, Bosa C, Pasanisi MB et al (2022) Acute and chronic synaptic pathology in multiple sclerosis gray matter. Mult Scler 28:369–382. 10.1177/13524585211022174 34124960 10.1177/13524585211022174

[214] Vertinsky AT, Li DKB, Vavasour IM, Miropolsky V, Zhao G, Zhao Y et al (2019) Diffusely abnormal white matter, T2 burden of disease, and brain volume in relapsing-remitting multiple sclerosis. J Neuroimaging 29:151–159. 10.1111/jon.12574 30376195 10.1111/jon.12574

[215] Volinsky R, Cwiklik L, Jurkiewicz P, Hof M, Jungwirth P, Kinnunen PKJ (2011) Oxidized phosphatidylcholines facilitate phospholipid flip-flop in liposomes. Biophys J 101:1376–1384. 10.1016/j.bpj.2011.07.051 21943418 10.1016/j.bpj.2011.07.051 PMC3177064

[216] Vrenken H, Seewann A, Knol DL, Polman CH, Barkhof F, Geurts JJG (2010) Diffusely abnormal white matter in progressive multiple sclerosis: in vivo quantitative MR imaging characterization and comparison between disease types. AJNR Am J Neuroradiol 31:541–548. 10.3174/ajnr.A1839 19850760 10.3174/ajnr.A1839 PMC7963986

[217] Waller R, Woodroofe MN, Wharton SB, Ince PG, Francese S, Heath PR et al (2016) Gene expression profiling of the astrocyte transcriptome in multiple sclerosis normal appearing white matter reveals a neuroprotective role. J Neuroimmunol 299:139–146. 10.1016/j.jneuroim.2016.09.010 27725112 10.1016/j.jneuroim.2016.09.010

[218] Watson C, Thirumalai D, Barlev A, Jones E, Bogdanovich S, Kresa-Reahl K (2023) Treatment patterns and unmet need for patients with progressive multiple sclerosis in the United States: survey results from 2016 to 2021. Neurol Ther 12:1961–1979. 10.1007/s40120-023-00532-2 37682512 10.1007/s40120-023-00532-2 PMC10630256

[219] Wegner C, Esiri MM, Chance SA, Palace J, Matthews PM (2006) Neocortical neuronal, synaptic, and glial loss in multiple sclerosis. Neurology 67:960–967. 10.1212/01.wnl.0000237551.26858.39 17000961 10.1212/01.wnl.0000237551.26858.39

[220] Werneburg S, Jung J, Kunjamma RB, Ha S-K, Luciano NJ, Willis CM et al (2020) Targeted complement inhibition at synapses prevents microglial synaptic engulfment and synapse loss in demyelinating disease. Immunity 52:167-182.e7. 10.1016/j.immuni.2019.12.004 31883839 10.1016/j.immuni.2019.12.004 PMC6996144

[221] Wheeler D, Bandaru VVR, Calabresi PA, Nath A, Haughey NJ (2008) A defect of sphingolipid metabolism modifies the properties of normal appearing white matter in multiple sclerosis. Brain 131:3092–3102. 10.1093/brain/awn190 18772223 10.1093/brain/awn190 PMC2577809

[222] Wheeler MA, Jaronen M, Covacu R, Zandee SEJ, Scalisi G, Rothhammer V et al (2019) Environmental control of astrocyte pathogenic activities in CNS inflammation. Cell 176:581-596.e18. 10.1016/j.cell.2018.12.012 30661753 10.1016/j.cell.2018.12.012 PMC6440749

[223] Wolswijk G, Balesar R (2003) Changes in the expression and localization of the paranodal protein Caspr on axons in chronic multiple sclerosis. Brain 126:1638–1649. 10.1093/brain/awg151 12805111 10.1093/brain/awg151

[224] Wong JK, Lin J, Kung NJ, Tse AL, Shimshak SJE, Roselle AK et al (2023) Cerebrospinal fluid immunoglobulins in primary progressive multiple sclerosis are pathogenic. Brain 146:1979–1992. 10.1093/brain/awad031 36732292 10.1093/brain/awad031 PMC10151187

[225] Wong WT (2013) Microglial aging in the healthy CNS: phenotypes, drivers, and rejuvenation. Front Cell Neurosci 7:22. 10.3389/fncel.2013.00022 23493481 10.3389/fncel.2013.00022 PMC3595516

[226] Woo MS, Engler JB, Friese MA (2024) The neuropathobiology of multiple sclerosis. Nat Rev Neurosci 25:493–513. 10.1038/s41583-024-00823-z 38789516 10.1038/s41583-024-00823-z

[227] Wood DD, Ackerley CA, van den Brand B, Zhang L, Raijmakers R, Mastronardi FG et al (2008) Myelin localization of peptidylarginine deiminases 2 and 4: comparison of PAD2 and PAD4 activities. Lab Invest 88:354–364. 10.1038/labinvest.3700748 18227806 10.1038/labinvest.3700748

[228] Xiao L, Ohayon D, McKenzie IA, Sinclair-Wilson A, Wright JL, Fudge AD et al (2016) Rapid production of new oligodendrocytes is required in the earliest stages of motor-skill learning. Nat Neurosci 19:1210–1217. 10.1038/nn.4351 27455109 10.1038/nn.4351 PMC5008443

[229] Ximerakis M, Lipnick SL, Innes BT, Simmons SK, Adiconis X, Dionne D et al (2019) Single-cell transcriptomic profiling of the aging mouse brain. Nat Neurosci 22:1696–1708. 10.1038/s41593-019-0491-3 31551601 10.1038/s41593-019-0491-3

[230] Yang L, Tan D, Piao H (2016) Myelin Basic Protein citrullination in multiple sclerosis: a potential therapeutic target for the pathology. Neurochem Res 41:1845–1856. 10.1007/s11064-016-1920-2 27097548 10.1007/s11064-016-1920-2

[231] Yates RL, Esiri MM, Palace J, Mittal A, DeLuca GC (2015) The influence of *HLA-DRB1*15* on motor cortical pathology in multiple sclerosis. Neuropathol Appl Neurobiol 41:371–384. 10.1111/nan.12165 24964187 10.1111/nan.12165

[232] Yates RL, Pansieri J, Li Q, Bell JS, Yee SA, Palace J et al (2022) The influence of *HLA-DRB1*15* on the relationship between microglia and neurons in multiple sclerosis normal appearing cortical grey matter. Brain Pathol 32:e13041. 10.1111/bpa.13041 34904300 10.1111/bpa.13041 PMC9245937

[233] Yeung MSY, Djelloul M, Steiner E, Bernard S, Salehpour M, Possnert G et al (2019) Dynamics of oligodendrocyte generation in multiple sclerosis. Nature 566:538–542. 10.1038/s41586-018-0842-3 30675058 10.1038/s41586-018-0842-3 PMC6420067

[234] Zeis T, Allaman I, Gentner M, Schroder K, Tschopp J, Magistretti PJ et al (2015) Metabolic gene expression changes in astrocytes in Multiple Sclerosis cerebral cortex are indicative of immune-mediated signaling. Brain Behav Immun 48:313–325. 10.1016/j.bbi.2015.04.013 25937052 10.1016/j.bbi.2015.04.013

[235] Zoupi L, Booker SA, Eigel D, Werner C, Kind PC, Spires-Jones TL et al (2021) Selective vulnerability of inhibitory networks in multiple sclerosis. Acta Neuropathol 141:415–429. 10.1007/s00401-020-02258-z 33449171 10.1007/s00401-020-02258-z PMC7882577

[236] Zrzavy T, Hametner S, Wimmer I, Butovsky O, Weiner HL, Lassmann H (2017) Loss of ‘homeostatic’ microglia and patterns of their activation in active multiple sclerosis. Brain 140:1900–1913. 10.1093/brain/awx113 28541408 10.1093/brain/awx113 PMC6057548

[237] Press Release: Tolebrutinib meets primary endpoint in HERCULES phase 3 study, the first and only to show reduction in disability accumulation in non-relapsing secondary progressive multiple sclerosis. https://www.sanofi.com/en/media-room/press-releases/2024/2024-09-02-05-00-00-2938875. Accessed 2 Jan 2025

